# Supplementary material for: QTL study reveals candidate genes underlying host resistance in a Red Queen model system
Source: PLoS Genet. 2023 Feb 2;19(2):e1010570. doi: 10.1371/journal.pgen.1010570 (PMC9894429; doi:10.1371/journal.pgen.1010570)
Supplement: S1 Methods — (DOCX) [file pgen.1010570.s001.docx]

## Supplementary materials and methods

### Infection experiment

We used an arbitrary subset of 40 F2 clones (20 attachment positive, and 20 attachment negative) from the QTL panel to test whether P21 hindgut attachment predicts infection outcome (S1 File). Thirty *Daphnia magna* individuals from each clone were housed in three separate 100-mL jars (ten individuals per jar) and exposed to 5,000 spores of the *Pasteuria ramosa* genotype P21 per day for three days (total 15,000 spores per individual). Animals were fed daily with 2 million cells of algae (*Tetradesmus obliquus*) until 10 days post infection, after which they were fed 10 million cells per day until the end of the experiment. Five weeks after infection, we scored all individuals for presence of diseases symptoms (empty brood pouch, red coloration), and a sample of individuals were also crushed and examined under a binocular microscope to confirm the presence of *P. ramosa* spores.

### QTL mapping

##### Phenotyping panel

We phenotyped all clones in the F2 panel using the attachment test [1]. Isolate P21 attaches to the hindgut of its host, so clones were considered susceptible if fluorescent spores were visible in the hindgut, and otherwise they were considered resistant. At least four individuals were scored for each clone. For clones with ambiguous results, additional individuals were scored.

##### Mapping with R/qtl

Phenotype data were combined with the genetic map data and analyzed using the R package qtl (version 1.42-8) [2]. Single QTL interval mapping was performed with a binary model. LOD significance thresholds were determined using permutation tests (1,000 permutations), and upper confidence limits on true p-values were determined using a binomial test. The QTL location (in cM) was estimated using a LOD support interval of 1.8, which is recommended for QTL panels created from an intercross.

A further genome scan using the QTL marker as an interactive covariate identified an additional QTL on linkage group 4 (position 20.4 cM; LOD 4.68; p-value 0.033) that may interact with the main effect QTL on linkage group 3. However, a 2-dimensional scan using a 2-QTL model failed to identify any linked loci or loci with marginal effects, and both manual and automated model search methods confirmed that the model with the single QTL at linkage group 3 had the maximum penalized LOD score.

### Fine mapping

##### Concept

Fine mapping is simply QTL mapping on a finer scale, and thus both methods rely on the same logic. QTL mapping works by crossing the parents (which differ in a phenotype of interest) and then selfing the resulting F1 generation to produce an F2 generation with random recombination events between the parental genotypes throughout the genome. By genotyping the F2 panel at genetic markers throughout the genome, the phenotype of interest can be associated with a genotype at a certain locus (under the assumption that F2 clones with the phenotype of interest have the same genotype as the parent clone with that phenotype). The goal of fine mapping, then, is to increase the resolution of the genetic map in the specific region that encompasses the QTL. This is accomplished by adding genetic markers within the QTL region and genotyping these markers in the informative clones from the QTL (F2) panel. The informative clones are those which show a phenotype-altering recombination event within the QTL region (i.e., in this study, such informative clones are expected to be susceptible to P21 attachment if the F locus is on one side of the recombination breakpoint, and resistant if the F locus is on the other side of the breakpoint). Using these informative clones, the QTL region can then be narrowed down by associating the phenotype to the matching genotype (based on the genotype/phenotype combination of the parent clones). The fine mapping has reached its limit when there are no more recombination events between the two markers, as these recombination “breakpoints” delimit the boundaries of the locus (for the particular collection of F2 clones). The ideal scenario is that the recombination events happen to be spaced such that the QTL can be fine mapped to the level of the gene which underlies the phenotype of interest. In this case, some clones must have a recombination event just upstream of the gene, and others must have a recombination event just downstream of the gene.

##### Experimental design

From the QTL analysis results, we narrowed down the region of interest to approximately 426 kb (Fig 1A), between the SNP markers scaffold00288_965 and scaffold01464_326 of the QTL map (S1 File, sheet “QTL SNP map”). Additional genotype data from a previous fine mapping effort [3] allowed us to reduce the region further to 350 kb (using the downstream flanking marker P24). We then began fine mapping using microsatellite analysis. We started by re-sequencing all the informative F2 clones (those which had previously shown a phenotype-altering recombination event in the region of interest [4]) at flanking markers P24 and 965D1 (just 165 bp downstream of previously used marker scaffold00288_965) in order to check the recorded genotypes of these clones (S1 File, sheet “Marker primers”). According to our results, only 18 of the 28 informative clones showed recombination events in the 350 kb region (S1 File, sheet “Finemap microsat”). We repeated the PCRs and sequencing analysis a second time and got the same results for all clones. We also sequenced the 28 F2 clones at markers from both ends of the ABC region (markers P34new and U1 (5.3 kb upstream of the forward primer from marker g311b that was previously used to delimit the ABC region [3])), to determine whether our locus of interest is upstream or downstream of this region. Lastly, we placed two additional markers on each side of the ABC region (markers 44154 and 90815 upstream and markers D16 and D42 downstream) to narrow the region down even further.

This first round of microsatellite sequencing (S1 File, sheet “Finemap microsat”) narrowed down the locus to a 58-kb region just downstream of (and overlapping the last 5.3 kb of) the ABC region (between markers U1 and D42), with just two informative clones remaining (clones 177 and 693). Clone 693 provides information on the upstream boundary of the locus, with a recombination breakpoint in the 5.3 kb between markers U1 and g311b (S1 File, sheet “Marker primers”). Clone 177 provides information on the downstream boundary, with a recombination breakpoint in the 25.7 kb between markers D16 and D42.

We next used Sanger sequencing for further fine mapping (S1 File, sheet “Finemap Sanger”). We re-sequenced clone 693 at marker g311b to confirm the location of the upstream boundary, and we sequenced clone 177 at two additional markers (D22 and D32) between D16 and D42 to narrow in on the downstream boundary. This reduced the locus to 40.4 kb, with the downstream boundary in the 8 kb between markers D16 and D22. In the second round of Sanger sequencing, we placed two additional markers (D16_5 and D19) between markers D16 and D22, which reduced the window of the downstream boundary from 8 kb to 6 kb. We also placed three additional markers (U0_5, U0_6, and U0_7) between markers U1 and g311b, which reduced the window of the upstream boundary from 5.3 kb to 2.5 kb. In the third and final round of Sanger sequencing, we placed 3 additional markers (D16_9, D17_1, D18) between markers D16_5 and D19, and we placed 2 additional markers (U0_55 and U0_54) between markers U0_5 and U0_6. The upstream recombination breakpoint was mapped to within 116 bp downstream of the SNP at position 2,329,948 (contig 000011F) in marker U0_55, and the downstream breakpoint was mapped to within 70 upstream of the SNP at position 2,361,178 (also contig 000011F) in marker D16_9.

Later evidence from F3 clones (see “Producing Selfed Offspring” below) allowed us to determine that the F locus is upstream of marker D16 (at position 2,358,729), thus narrowing the region by another 2.4 kb.

### Marker creation

##### Microsatellite markers

Size-polymorphic markers were created for analysis with capillary electrophoresis (S1 File, sheet “Marker primers”). These markers are similar to microsatellites in that they are size polymorphic between the two parent clones, and the target region is smaller than that for SNP markers, which are sequenced with Sanger sequencing. We aligned the F-locus region (between markers scaffold00288_965 and P24) of the parent clones Xinb3 and Iinb1 in MEGA (version X) [5] using ClustalW alignment [6]. We then manually searched for size polymorphisms (gaps in alignment) between the two clones in the genomic areas where we wanted to place our markers. Only gaps larger than 3 bp were considered in order to minimize the risk of including spurious gaps that were the result of assembly errors (both genomes were sequenced using PacBio technology, which is susceptible to such errors). A 400-bp region surrounding each gap was searched for primers using Primer3Web (version 4.1.0) [7,8], and we checked that the primer pairs were identical between the parent clones. Target sizes varied from 142 bp to 339 bp. Primer pairs were also checked for mis-priming using BLASTN (version 2.7.1+, blast.ncbi.nlm.nih.gov) against the Xinb3 reference genome version 3.0. We used the program Multiple Primer Analyzer (ThermoFisher) to check for possible cross-primer dimers in order to optimize the primer pair combinations in each of two multiplex runs. Unlabeled primers were used to optimize PCR conditions, and 5’ fluorescently-labeled forward primers were used for capillary electrophoresis.

SNP markers for Sanger Sequencing

Sanger sequencing-appropriate primers were created to amplify regions containing at least one SNP between the QTL parent clones (S1 File, sheet “Marker primers”). We searched a region of approximately 1 kb for primers with product sizes between 150 and 700 bp. Chosen product sizes ranged from 128 bp to 557 bp. QTL F2 panel clones 693 or 177 were sequenced at each of the created markers, and sequences were compared to those of the QTL parent clones (Xinb3 and Iinb1) to determine the genotype at each marker position.

##### Marker sequencing

DNA was extracted from whole *Daphnia magna* using Chelex beads (Bio-Rad), as adapted from Walsh et al. [9]. Individual *D. magna* were placed in 96-well plates with a drop of water and crushed using plastic pestles. To each well was added 150 µL of 5-10 % (w/v) Chelex beads and 10 µL of 20 % (w/v) proteinase K, and samples were incubated for 2 hours at 55 °C followed by 10 minutes at 99 °C. Sample plates were centrifuged for 5 minutes at 7,000 rpm to separate DNA from the Chelex beads, and 75-100 µL of the supernatant was removed for PCR amplification.

For the first round of fine mapping, we used a microsatellite sequencing protocol as described in Andras and Ebert [10] to amplify markers that were size polymorphic between the QTL parents Xinb3 and Iinb1. Forward primers from eight primer pairs were uniquely labeled with fluorescent dyes and combined in multiplex PCR reactions (two runs of four primers). Primers, DNA, and master mix (2x Qiagen Multiplex PCR Master Mix) were combined and heated to 95°C for 15 minutes to activate the Taq polymerase. This initial heating was followed by 30 PCR cycles as follows: 30 seconds at 94°C (denature), 90 seconds at 60°C (anneal), 90 seconds at 72°C (extend). Optimal annealing temperature had been determined previously using a gradient PCR for all primer pairs. After all PCR cycles were complete, samples were incubated for 10 minutes at 72°C for the final extension. PCR products were added to a microsatellite master mix (containing Applied Biosystems Hi-Di formamide and GeneScan500LIZ dye size standard) and incubated for 4 min at 94°C and then snap cooled on ice for 4 minutes. The PCR product was then analyzed with capillary electrophoresis (Applied Biosystems 3130xl Genetic Analyzer), and the resulting electropherogram peaks were interpreted using GeneMapper Software (version 4.1, Applied Biosystems) to distinguish the homozygotes and the heterozygotes.

For the second and third rounds of fine mapping, PCRs were performed as previously described, but with a separate reaction for each primer pair, and with an annealing temperature of 58°C (again determined with gradient PCR for this new set of primers). PCR products were run on an agarose gel to confirm the presence of sufficient DNA for sequencing. PCR products were then sent for Sanger Sequencing (Microsynth; Basel, Switzerland). Chromatogram results were examined visually for sequencing accuracy and to check for overlapping peaks in the case of potential heterozygotes.

### Producing selfed offspring

We produced F3 offspring from clones 693 and 94 of the QTL F2 panel, primarily to validate that the F locus and ABC supergene are separate loci, and secondarily to further refine the interval of the F-locus region and to check for possible epistasis between the D and F loci (S2 File). We chose F2 clones 693 and 94 because they were predicted to have experienced recombination between the F locus and the ABC supergene (recombination is necessary to demonstrate that the two loci are different) and they were also potentially heterozygous at the F locus (heterozygosity is necessary to produce segregating offspring that can be used for further fine mapping). Selfed offspring were produced by collecting and hatching the sexually-produced ephippia (resting eggs) from single-clone jars of these F2 clones (S2 File, sheet “Collection”). To stimulate ephippia production, we placed the animals in modified laboratory conditions, with a lower temperature (18 °C instead of 20 °C) and shorter day (8:16 hour light/dark instead of 18:6 hour light/dark). Ephippia were collected four times at approximately two-week intervals. Collected ephippia were placed in 1.5 mL plastic tubes and stored in the dark at 4 °C for at least six weeks to simulate winter. After this dormancy period, ephippia were dried by placing them on suspended coffee filters in a well-aerated room for 4 – 5 days. Then ephippia were stimulated to hatch by first placing them in a 50 % bleach solution (in deionized water) for four minutes, then rinsing thoroughly in deionized water for one minute and placing them in artificial Daphnia medium (ADaM) [11,12] until hatching (after approximately 5 – 10 days). Newly hatched offspring were transferred individually to new 100 mL jars and cared for under standard laboratory conditions [12].

All 74 F3 offspring of F2 clone 94 were resistant to P21 attachment, strongly suggesting that clone 94 is homozygous dominant (genotype FF) and indicating that the F locus is upstream of marker D16 (because clone 94 is heterozygous at this marker). In contrast, the F3 offspring of F2 clone 693 segregated for P21 resistance: 8 out of 29 (28%) offspring tested were susceptible to P21 attachment. This strongly suggests that clone 693 is heterozygous at the F locus and confirms that the F and C loci are separate, because clone 693 is homozygous recessive at the C locus, which was inferred previously [3] and was supported by our result showing that 22 out of 22 (100%) of F3 offspring from clone 693 were susceptible to C19 attachment. Two out of 22 (8 %) of the F3 offspring from F2 clone 693 were susceptible to P15 hindgut attachment, although the D-locus genotype (dd) of clone 693 predicted zero susceptible offspring. This slight distortion of segregation suggests that the F locus could have an effect on P15 attachment.

### Cladoceran-specific protein family

The Cladoceran-specific (CS) proteins described here form a large family of molecules (over 100 members detected so far), the genes of which are distributed in multiple locations of the *D. magna* genome. These include gene islets linked to *Pasteuria ramosa* resistance loci ABC and F (located on contig 000011F (2991638 bp) in the Xinb3 reference genome version 3.0) as well as to the D locus (located on contig 000018F (2589983 bp) in the Xinb3 reference genome version 3.0), which we aligned using Clustal Omega [13] (S1 Fig). Some of the CS proteins are membrane bound, thanks to a hydrophobic segment. Many others are susceptible to be secreted. As their name suggests, homologs of these genes have so far been found only in Cladocerans.

The sequence of a CS (one example below) shows some highly conserved features:

- An average predicted sequence length of about 190 – 300 amino acids (with a few exceptions reaching up to 1148 aa)
- A high percentage of amino acid identities (from 35 – 70 %)
- A richness in T and S residues, suggesting a high level of glycosylation
- Some (4) highly conserved cysteine residues at positions 442 – 472 and 708 – 718 (see sequence below and S1 Fig)
- Some highly conserved charged residues (K and R) at positions 473 – 483 (S1 Fig)

>11F23.93 CS

MEFTFILLSALVAVSQQQFRRHPSEGMFWLASYYSPPSATINPYLTSNYNNDELVVMPFFRQLPRDDPDVEEIFGGTQLRNKEMNRYQPFRQDKARLVVNFSSRSYLLNKVKTISFTITSSVTLTKVES**C**IPSHQFSASFASVT**C**RRKRGGIAELPVTNWKDIQLATKPTNVQPVEPTIVSALNSPTGSAELPQISSSKDEDLLNEKQSSQVIPPSQQARMKRLLFHFVATTTVVSYTFFSATSTKTVSLLSVADQGPGFLICRPEGYSVCS

### Gene annotation

Software-generated annotations were manually curated using a combination of transcriptomic data, protein structure predictions, and alignment to homologous genes (S6 File). Briefly, RNAseq reads sequenced previously [14] were assembled *de novo* to their respective genomes using TRINITY (version 2.11.0) [15] and rnaSPADES (version 3.14.1) [16]. Each sample was assembled as an independent transcriptome, and then all these transcriptomes were combined and de-duplicated to produce a consensus transcriptome for each QTL parent clone (Xinb3 and Iinb1). We then mapped the resulting transcripts back to their respective genome using minimap2 (version 2.17-r941) [17] with the splice:hq option (long-read splice alignment for PacBio CCS reads), filtering out transcripts with bit flag 0x100 (secondary alignments) and mapQ score below 60 to retain only uniquely mapped transcripts. We then used the mapped transcripts to curate the structural gene annotations. For example, sometimes two neighboring genes were found to have transcripts spanning both sequences, suggesting that they should be instead annotated as a single gene. Also, many genes did not have their flanking untranslated regions (UTRs) annotated, so in some cases we could use the transcripts to add these UTR annotations. This hand curation was also important for making accurate comparisons between homologous genes in xF and iF (e.g., for percent identity and piN/piS calculations).

A summary of the manual curations for each QTL parent genome is listed below (see S6 File for additional details):

##### Xinb3 genes (all on contig 000011F)

**23.92**: extended 3’ UTR by 183 bp to match transcript contig71687.1

**23.84**: extended 5’ UTR by 24 bp to match transcript contig153863.1. I did this for both mRNA-1 and mRNA-2 because the transcript evidence does not favor one over the other.

**23.20**: annotated 5’ UTR based on transcript contig173441.1 and annotated 3’ UTR based on transcript contig140187.1

**23.8**: extended 5’ UTR by 7 bp to match transcript contig149714.1

**23.93**: annotated 3’ UTR based on transcript contig188817.1

**23.73**: made 5’ end of gene match the annotation for Iinb1 homolog 2592. This involved joining the two 5’ exons and making them 5’ UTR rather than CDS. Evidence for changes comes from alignment with Iinb1 homolog and other paralogs in F locus: protein alignment shows unusually long leader and unusually short first CDS.

**23.23**: annotated 5’ UTR based on transcript contig110083.1

**23.10**: annotated 5’ UTR based on transcript contig115927.1

##### Iinb1 genes (all on contig 129)

**2589**: Annotated both UTRs based on transcript contig16355.1

**2590**: Annotated 3’ UTR based on transcript contig14710.1

**2591**: Extended second CDS by 8 bp based on transcript contig36462.1 and alignment with Xinb3 homolog 23.93. This caused premature stop codon in protein sequence, so next CDS shortened and 3’ UTR lengthened accordingly.

**2593**: Combined genes 2593 and 2594 into one single gene based on transcript evidence, alignment to Xinb3 homolog 23.9, and predicted functional annotation as VEGF receptor. Shifted some exons and added one exon based on transcript contig5457.1. Shifted 5’ CDS and added 5’ UTR based on transcripts contig5353.1 and contig49933.1. Changed last seven exons into 3’ UTR based on a 1-bp deletion (relative to transcripts and Xinb3 reference) between positions 728968 and 728969 in the Iinb1 reference sequence that caused a frame shift and premature stop codon in the reference haplotype.

**2603**: Removed two 5’ CDS regions based on protein structure prediction (leader is unusually long) as well as alignment to Xinb3 homolog 23.53 and to the other Cladoceran-specific paralogs (see S4 Fig).

Besides using the transcript evidence to curate the structural annotations, we also refined the functional annotations by examining the translated protein structures using a combination of programs including INTERPROSCAN (version 5.39-77.0) [18], PROSITE (version 2019_11) [19], SWISS-MODEL (version 2021-12-17) [20,21], and SMART (version 8.0) [22]. These methods revealed that gene 23.92 is similar to a lactosylceramide 4-alpha-galactosyltransferase (previously annotated as an alpha(1,3)fucosyltransferase) and that gene 23.9 is likely a VEGF receptor (previously annotated as brain chitinase and chia).

## References

1. Duneau D, Luijckx P, Ben-Ami F, Laforsch C, Ebert D. Resolving the infection process reveals striking differences in the contribution of environment, genetics and phylogeny to host-parasite interactions. BMC biology. 2011;9:11.

2. Broman KW, Wu H, Sen S, Churchill GA. R/qtl: QTL mapping in experimental crosses. Bioinformatics. 2003;19:889–90.

3. Bento G, Routtu J, Fields P, Bourgeois Y, Du Pasquier L, Ebert D. The genetic basis of resistance and matching-allele interactions of a host-parasite system: The *Daphnia magna-Pasteuria ramosa* model. PLOS Genetics. 2017;13.

4. Routtu J, Hall MD, Albere B, Beisel C, Bergeron RD, Chaturvedi A, et al. An SNP-based second-generation genetic map of *Daphnia magna* and its application to QTL analysis of phenotypic traits. BMC Genomics. 2014;15:1033.

5. Kumar S, Stecher G, Li M, Knyaz C, Tamura K. MEGA X: Molecular Evolutionary Genetics Analysis across computing platforms. Molecular Biology and Evolution. 2018;35:1547–9.

6. Thompson JD, Gibson TJ, Higgins DG. Multiple sequence alignment using ClustalW and ClustalX. Current protocols in bioinformatics. 2003;2–3.

7. Untergasser A, Cutcutache I, Koressaar T, Ye J, Faircloth BC, Remm M, et al. Primer3—new capabilities and interfaces. Nucleic Acids Research. 2012;40:e115.

8. Koressaar T, Remm M. Enhancements and modifications of primer design program Primer3. Bioinformatics. 2007;23:1289–91.

9. Walsh PS, Metzger DA, Higuchi R. Chelex 100 as a medium for simple extraction of DNA for PCR-based typing from forensic material. BioTechniques. 1991;10:506–13.

10. Andras JP, Ebert D. A novel approach to parasite population genetics: Experimental infection reveals geographic differentiation, recombination and host‐mediated population structure in *Pasteuria ramosa*, a bacterial parasite of *Daphnia*. Molecular Ecology. 2013;22:972–86.

11. Klüttgen B, Dülmer U, Engels M, Ratte HT. ADaM, an artificial freshwater for the culture of zooplankton. Water Research. 1994;28:743–6.

12. Ebert D, Zschokke-Rohringer CD, Carius HJ. Within- and between-population variation for resistance of *Daphnia magna* to the bacterial endoparasite *Pasteuria ramosa*. Proceedings of the Royal Society B: Biological Sciences. 1998;265:2127–34.

13. Madeira F, Pearce M, Tivey ARN, Basutkar P, Lee J, Edbali O, et al. Search and sequence analysis tools services from EMBL-EBI in 2022. Nucleic acids research. 2022;gkac240.

14. Orsini L, Gilbert D, Podicheti R, Jansen M, Brown JB, Solari OS, et al. *Daphnia magna* transcriptome by RNA-Seq across 12 environmental stressors. Scientific Data. 2016;3:160030.

15. Grabherr MG, Haas BJ, Yassour M, Levin JZ, Thompson DA, Amit I, et al. Full-length transcriptome assembly from RNA-Seq data without a reference genome. Nature biotechnology. 2011;29:644–52.

16. Bushmanova E, Antipov D, Lapidus A, Prjibelski AD. rnaSPAdes: a de novo transcriptome assembler and its application to RNA-Seq data. GigaScience. 2019;8:giz100.

17. Li H. Minimap2: pairwise alignment for nucleotide sequences. Bioinformatics. 2018;34:3094–100.

18. Apweiler R, Attwood TK, Bairoch A, Bateman A, Birney E, Biswas M, et al. The InterPro database, an integrated documentation resource for protein families, domains and functional sites. Nucleic Acids Research. 2001;29:37–40.

19. Sigrist CJ, Cerutti L, Hulo N, Gattiker A, Falquet L, Pagni M, et al. PROSITE: a documented database using patterns and profiles as motif descriptors. Briefings in bioinformatics. 2002;3:265–74.

20. Waterhouse A, Bertoni M, Bienert S, Studer G, Tauriello G, Gumienny R, et al. SWISS-MODEL: homology modelling of protein structures and complexes. Nucleic Acids Research. 2018;46:W296–303.

21. Guex N, Peitsch MC, Schwede T. Automated comparative protein structure modeling with SWISS-MODEL and Swiss-PdbViewer: a historical perspective. Electrophoresis. 2009;30 Suppl 1:S162-173.

22. Letunic I, Bork P. 20 years of the SMART protein domain annotation resource. Nucleic Acids Research. 2018;46:D493–6.
