# Supplementary material for: QTL study reveals candidate genes underlying host resistance in a Red Queen model system
Source: PLoS Genet. 2023 Feb 2;19(2):e1010570. doi: 10.1371/journal.pgen.1010570 (PMC9894429; doi:10.1371/journal.pgen.1010570)
Supplement: S1 Fig — Clustal Omega alignment of amino acid sequences from 53 Cladoceran-specific genes located on contigs 000011F (containing the ABC supergene and the F locus) and 000018F (containing the D locus). cov: coverage; pid: percent identity. Residues of note: cysteine: yellow; charged amino acids: red; serine and threonine: light blue. Consensus sequence is indicated below the alignment and is particularly notable between positions 400 and 720. (HTML) [file pgen.1010570.s002.html]

MView


|  |
| --- |
| ``` Reference sequence (1): 18F-16.50(2) Identities normalised by aligned length. Colored by: identity ``` |
| ```                      cov    pid    1 [        .         .         .         .         :         .         .         . 80    1 18F-16.50(2)   100.0% 100.0%      -------------MIVRVPLLS-----------------------AVPVFM--------AVCLVL-TAKQDVQV----AH       2 18F-15.31       67.1%  21.0%      --------------------------------------------------------------------------------       3 18F-16.61       64.3%  25.1%      --------------------------------------------------------------------------------       4 11F-16.38       66.6%  21.1%      ----------------------------------------------------------------M-QF------------       5 18F-16.117(1)   59.2%  22.1%      ----------------------------------------------------------------M-HYVPHRPV----ID       6 18F-16.117(2)   61.5%  30.0%      --------------------------------------------------------------------------------       7 18F-16.77       59.5%  20.7%      --------------------------------------------------------------------------------       8 18F-16.102      57.8%  17.9%      --------------------------------------------------------------------------------       9 18F-16.116      48.7%  11.5%      --------------------------------------------------------------------------------      10 18F-16.118      65.2%  12.9%      ---------------------------------------------MVPHTFFVKRVDVDAP-----GNSEVLNT----VI      11 18F-24.234      47.3%  14.1%      --------------------------------------------------------------------------------      12 18F-21-123      47.0%  13.6%      --------------------------------------------------------------------------------      13 18F-21.121(1)   45.3%  12.4%      --------------------------------------------------------------------------------      14 18F-21.122      46.7%  13.7%      --------------------------------------------------------------------------------      15 18F-16.50(1)    45.3%  11.5%      --------------------------------------------------------------------------------      16 18F-16.79       41.4%   7.9%      --------------------------------------------------------------------------------      17 18F-16.153      48.4%   9.0%      --------------------------------------------------------------------------------      18 18F-12.31       49.0%  12.2%      --------------------------------------------------------------------------------      19 11F-17.59       51.3%  11.6%      --------------------------------------------------------------------------------      20 18F-17.59       51.3%  11.6%      --------------------------------------------------------------------------------      21 18F-18.10       75.6%   9.1%      -------MKSILQLIIVMQLFTMLLVDGQRTSTYTESRTISTTVDFTSNLICVKLVNVTGRCRQQRGFASEQPI----IL      22 18F-18.11       67.1%  10.9%      --------------------------------------------------------------------------------      23 18F-22.75       66.6%  12.5%      --------------------------------------------------------------------------------      24 18F-10.79(1)    52.7%   5.9%      -------MKLS---LMFIVGC--ITASL---------------------------------------SYGQPPF------      25 18F-10.50       47.0%   7.9%      -------MKI----ALVLVVC--FVS-L---------------------------------------TLQQN-V------      26 18F-10.71       46.7%   7.9%      -------MKI----ALVLFAC--FVS-L---------------------------------------TLQQN-V------      27 18F-10.68       43.6%   6.9%      MTSDNNVMKL----ALLVLGC--FVC-L---------------------------------------TKQER-Y------      28 18F-10.69.1     49.0%   5.9%      -------MKLF---LSILLFC--FVS-V---------------------------------------SYQEN-N------      29 18F-10.69.2     38.2%   7.2%      --------------------------------------------------------------------------------      30 18F-13.28(2)    56.9%   8.7%      -------MKFL----VLVLAV--AVVAGN-------------------------------------IFHRPFSTQ--LKP      31 11F-23.10       55.2%   7.1%      -------MKLSVFLLIL------VAA-----------------------------------------SHQQYLGRNMFRL      32 11F-2.50        54.7%   8.1%      -------MKIFLGTMVLVSAV--ISIYPQ-------------------------------------HFHQHSVGQY---R      33 11F-2.51        57.8%   7.4%      -------MKFVFGLILLVSAL--VAISPQ-------------------------------------QFHQQRTGQ-----      34 11F-13.72(1)    61.2%   8.6%      -------MKLA---LVFLMAL--VVISQQ-------------------------------------FYLR-QPY---SRT      35 11F-13.72(2)    70.5%   8.2%      -------MKIG---ILLLLAL--VVISQQ-------------------------------------FYLR-QPY---SRA      36 11F-23.23       43.6%  10.1%      --------------------------------------------------------------------------------      37 11F-23.73       54.4%   8.8%      ------MVKFA---LVLLLGL--VAISNQ-------------------------------------QFYQQPAS---ERL      38 11F-23.98       54.1%   9.5%      -------MKFA---LAFLLGL--VVISNQ-------------------------------------QFHQQPTN---DRI      39 11F-23.53       51.6%   6.5%      -------MKFA---LISLLSV--VVFSNQ-------------------------------------QFHEQSTD---EKL      40 11F23.93        50.7%   7.5%      -------MEFT---FILLSAL--VAVSQQ-------------------------------------QFRR-HPS---EGM      41 11F-22.90(1)    43.1%   6.5%      -------MKLT---LVFVTIC--VAFSQQ-------------------------------------QYYHPRMI---MGY      42 11F-22.90(2)    52.1%   6.7%      -------MKLT---LLFLSVC--VAISHQ-------------------------------------QYYRSHMI---VSN      43 18F-13.28(1)    53.0%   9.4%      -------MKLA---LICLSIL--LAVSCS-------------------------------------QQRL---N---KRL      44 11F-24.30(3)    62.6%   6.6%      -------MKFV---LIILISV--LVVGTH-------------------------------------QYFH-HRP---REM      45 11f-0.66(2)     39.1%   6.9%      -------MKLA---LIFLFAA--VVITNQ-------------------------------------Q-FQ--QQ---REK      46 11F-0.66(1)     53.0%   5.8%      -------MKFA---LIFLSAL--VAITHQ-------------------------------------QFQQ--QH---MGR      47 11F-0.66(3)     51.3%   8.2%      -------MKLA---LIFLSVF--LVAFSD-------------------------------------QQFQ--QR---RER      48 11F-24.30(2)    59.2%   6.1%      -------MKFV---LIFLGSV--LVVGTH-------------------------------------QYFD--DQ---RGE      49 11F-21.42       54.4%   8.9%      -------MKFT---LVFLCAC---VVITH-------------------------------------QQFQ--RP---RAM      50 11F-24.30(1)    49.0%   7.1%      -------MKFA---FIFLSTL---MVMSH-------------------------------------QQLQ--RT---RVV      51 11F.26.28       51.8%   4.7%      -------MKFA---LLFLSAL---VVLSH-------------------------------------QQFQ--HP---RGL      52 11F-24.30(4)    60.6%   3.4%      -------MKFV---FIFLMSV--VFAVSS-------------------------------------QQ-RAPRP---RTL      53 11F-24.30(6)    51.8%   6.7%      -------MKFA---LIFLLSA--VVALSQ-------------------------------------QQFLRPRP---HGL         consensus/100%                    ................................................................................         consensus/90%                     ................................................................................         consensus/80%                     ................................................................................         consensus/70%                     ................................................................................                            cov    pid   81          .         1         .         .         .         .         :         . 160   1 18F-16.50(2)   100.0% 100.0%      L-LTGYHPLSRLGILQQG-----PSRMMASKAMT-------------------------TYVVY--RT----T-------       2 18F-15.31       67.1%  21.0%      --------------------------------------------------------------------------------       3 18F-16.61       64.3%  25.1%      --------------------------------------------------------------------------------       4 11F-16.38       66.6%  21.1%      --------------------------------------------------------------------------------       5 18F-16.117(1)   59.2%  22.1%      L-FQNVQHIEAFGLFIKA-----DVR------------------------------------------------------       6 18F-16.117(2)   61.5%  30.0%      --------------------------------------------------------------------------------       7 18F-16.77       59.5%  20.7%      --------------------------------------------------------------------------------       8 18F-16.102      57.8%  17.9%      --------------------------------------------------------------------------------       9 18F-16.116      48.7%  11.5%      --------------------------------------------------------------------------------      10 18F-16.118      65.2%  12.9%      VAEE----KTDVR----------PSRSXXXXXXXX-----------XXXXXXX-----XXXXXXXXXXXXXXXXX-----      11 18F-24.234      47.3%  14.1%      --------------------------------------------------------------------------------      12 18F-21-123      47.0%  13.6%      --------------------------------------------------------------------------------      13 18F-21.121(1)   45.3%  12.4%      --------------------------------------------------------------------------------      14 18F-21.122      46.7%  13.7%      --------------------------------------------------------------------------------      15 18F-16.50(1)    45.3%  11.5%      --------------------------------------------------------------------------------      16 18F-16.79       41.4%   7.9%      --------------------------------------------------------------------------------      17 18F-16.153      48.4%   9.0%      --------------------------------------------------------------------------------      18 18F-12.31       49.0%  12.2%      --------------------------------------------------------------------------------      19 11F-17.59       51.3%  11.6%      --------------------------------------------------------------------------------      20 18F-17.59       51.3%  11.6%      --------------------------------------------------------------------------------      21 18F-18.10       75.6%   9.1%      MMDGPFHPLSQFM----------PTQTLRVEATPL-----------IVSSDWP-----IDYSFQT-PTNLEPSMP-----      22 18F-18.11       67.1%  10.9%      ---------------------------MKIIVVL----------------------------FMV-TLCVG---------      23 18F-22.75       66.6%  12.5%      ---------------------------MKLASFF--------------------------FILCL-ANFIA---------      24 18F-10.79(1)    52.7%   5.9%      QWQQMRP-AS-----RN-----RQFMTL------IDFRNSDADNVIADNDQW--------------DSADEPLWRQEDGG      25 18F-10.50       47.0%   7.9%      FWSYPGR-RV-------------PYYSM------YNHNIPDDLPIH-------------------------------AAL      26 18F-10.71       46.7%   7.9%      FWSFPGR-RN-------------PYYSM------FSHNAQDDLPIV-------------------------------AAL      27 18F-10.68       43.6%   6.9%      VWPF-P--ID-----PNH----AVYKPL------YYSD-----------------------------------------G      28 18F-10.69.1     49.0%   5.9%      VRPPVPY-AR-----QSR----ARYVPL------FYAN-----------------------------------------G      29 18F-10.69.2     38.2%   7.2%      -------------------------MPL------FYAD-----------------------------------------G      30 18F-13.28(2)    56.9%   8.7%      SWPIVSSPAYQHPFLSNKGSYYHQSQPKDCEEHQNYISFYDYFPFYPY------------------PTN---IQQGVYSG      31 11F-23.10       55.2%   7.1%      PWISTRF-AQQQPIEY----------PH------YYNPRYHD---DSYSGPDAT-------PVIIVNRVNE---------      32 11F-2.50        54.7%   8.1%      SWLPYYQ-QPRTEFQYGYE--YQPTALL------HYNPVAADLPSFRHSYPENT-------PTTFFREDEG-MGP-----      33 11F-2.51        57.8%   7.4%      QWLSQYH-QLPRTFLYNQ---RQPAASL------YYEPVDANIPFFRYSRPNIQ-------PALIYPQNEEGSYQGAFDG      34 11F-13.72(1)    61.2%   8.6%      FWLSPYP-PQDF--LSHY----Q---PL------PENLD-----------------------------------------      35 11F-13.72(2)    70.5%   8.2%      FWVSPYT-SPRV--ANNY----R---PL------IFITHEDFK-------------------------------------      36 11F-23.23       43.6%  10.1%      -----------------------------------------------------------------------MNSQ-----      37 11F-23.73       54.4%   8.8%      LWLLSYY-PPQAT-FNTY----N-YQHD------NNHDMGDGRASTFPNSLSHSLTI----P-------TYSQDK-----      38 11F-23.98       54.1%   9.5%      LWLLTYY-SPRPT-IRDY----N-YQPV------NYDVSEDVGPPTFFKQLTPSSKN----P-------IPSQVK-----      39 11F-23.53       51.6%   6.5%      LWLLSYY-SPQLP-VSKY----N-YQPV------NHHNSDDGNYHLRPSSSGSSMNSQSINDYLSSNYNNDEL-E-----      40 11F23.93        50.7%   7.5%      FWLASYY-SPPS---------------------------------------------ATINPYLTSNYNNDELVV-----      41 11F-22.90(1)    43.1%   6.5%      PWMS-PF-AHQPM-FDDY----VD-----------------RY------------------PYMV---------------      42 11F-22.90(2)    52.1%   6.7%      PWLLPPF-ARQPMFFNNY----MD-----------------HY------------------PYAG---------------      43 18F-13.28(1)    53.0%   9.4%      TWPISYY-SPRTFYPWPY---------S------LD-----DF------------------PLDSLNPSPLVSDE-----      44 11F-24.30(3)    62.6%   6.6%      PWVSPYL-PHSFIVYLTK----P------------------NP--------------PTSYP---TQSE-----------      45 11f-0.66(2)     39.1%   6.9%      LWWSPYF-SAE--RIINY----Q---PI------YYDPVQDEMQFFRQYRPM-R-------PIPYLQSEV----------      46 11F-0.66(1)     53.0%   5.8%      PWWLPSY-YSPQPTVSSY----Q---HI------YYNTFPENTPSFRQVRPS-R-------PYLTTNNAQ----------      47 11F-0.66(3)     51.3%   8.2%      LWWTPYN-YAQQPFVNNY----Q---ES------YYNV-QDDIPDYRVFRPT-R-------PVTYSQNDE----------      48 11F-24.30(2)    59.2%   6.1%      IRWASYQ-PHPFIE--KD----PPY--L------VYFSIKPNP--------------PTIYPLHDLLPENLAQDA-----      49 11F-21.42       54.4%   8.9%      LWLSPYY-SSPQSAVNNY----QAYRY--------ND-----VP---------------------YLND-----------      50 11F-24.30(1)    49.0%   7.1%      PMLSPYT-SFIRN-----------YHPL------YYDNAEDVMPYVRYSRPL-R---PT---IYSLENE-----------      51 11F.26.28       51.8%   4.7%      VWLSPYI-QSQQPILHQH------QQAL------YQHGSNEDVDGRALRRRY-R---PQSIPVSYLQNE-----------      52 11F-24.30(4)    60.6%   3.4%      VWVTPYS-PTHNPVLANY----Q---PV------YEHNIQDEIPTKLTPHSF-RRKHKPSIAASYFQNE-----------      53 11F-24.30(6)    51.8%   6.7%      VWWSPYL-HPQPASRNDF----HSGQSL------YDDVE--------------------------QENE-----------         consensus/100%                    ................................................................................         consensus/90%                     ................................................................................         consensus/80%                     ................................................................................         consensus/70%                     ................................................................................                            cov    pid  161          .         .         .         2         .         .         .         . 240   1 18F-16.50(2)   100.0% 100.0%      ------------------------------------------------------------------------IPTCSFT-       2 18F-15.31       67.1%  21.0%      --------------------------------------------------------------------------------       3 18F-16.61       64.3%  25.1%      --------------------------------------------------------------------------------       4 11F-16.38       66.6%  21.1%      --------------------------------------------------------------------------------       5 18F-16.117(1)   59.2%  22.1%      ------------------------------------------------------------------------------R-       6 18F-16.117(2)   61.5%  30.0%      --------------------------------------------------------------------------------       7 18F-16.77       59.5%  20.7%      --------------------------------------------------------------------------------       8 18F-16.102      57.8%  17.9%      --------------------------------------------------------------------------------       9 18F-16.116      48.7%  11.5%      --------------------------------------------------------------------------------      10 18F-16.118      65.2%  12.9%      --XXX----------------------------------------------------------------XXXXXXXXXX-      11 18F-24.234      47.3%  14.1%      --------------------------------------------------------------------------------      12 18F-21-123      47.0%  13.6%      --------------------------------------------------------------------------------      13 18F-21.121(1)   45.3%  12.4%      --------------------------------------------------------------------------------      14 18F-21.122      46.7%  13.7%      --------------------------------------------------------------------------------      15 18F-16.50(1)    45.3%  11.5%      --------------------------------------------------------------------------------      16 18F-16.79       41.4%   7.9%      --------------------------------------------------------------------------------      17 18F-16.153      48.4%   9.0%      --------------------------------------------------------------------------------      18 18F-12.31       49.0%  12.2%      --------------------------------------------------------------------------------      19 11F-17.59       51.3%  11.6%      --------------------------------------------------------------------------------      20 18F-17.59       51.3%  11.6%      --------------------------------------------------------------------------------      21 18F-18.10       75.6%   9.1%      --RTS----------------------------------------------------------------ARRKQLGYFS-      22 18F-18.11       67.1%  10.9%      ------------------------------------------------------------------------PSLCVWG-      23 18F-22.75       66.6%  12.5%      ------------------------------------------------------------------------PSSCFWS-      24 18F-10.79(1)    52.7%   5.9%      VPHLR----------------------------------------------------------------------QYHHN      25 18F-10.50       47.0%   7.9%      DPAGH---------------------------------------------------------------------------      26 18F-10.71       46.7%   7.9%      DPAGH---------------------------------------------------------------------------      27 18F-10.68       43.6%   6.9%      LPAGY---------------------------------------------------------------------------      28 18F-10.69.1     49.0%   5.9%      VPAGY---------------------------------------------------------------------------      29 18F-10.69.2     38.2%   7.2%      QPAAD---------------------------------------------------------------------------      30 18F-13.28(2)    56.9%   8.7%      QPRSP---------------------------------------------------------------------------      31 11F-23.10       55.2%   7.1%      --------------------------------------------------------------------------------      32 11F-2.50        54.7%   8.1%      --------------------------------------------------------------------------------      33 11F-2.51        57.8%   7.4%      IPQYYIDNKQSVQQKNQDLLLKQVGQDNEEKKAFTVFPYVRPIAPSAVYSQADGDESIIELLASHNLFADGPSKVGYFRP      34 11F-13.72(1)    61.2%   8.6%      --------------------------------------------------------------------------------      35 11F-13.72(2)    70.5%   8.2%      --------------------------------------------------------------------------------      36 11F-23.23       43.6%  10.1%      --------------------------------------------------------------------------------      37 11F-23.73       54.4%   8.8%      --------------------------------------------------------------------------------      38 11F-23.98       54.1%   9.5%      --------------------------------------------------------------------------------      39 11F-23.53       51.6%   6.5%      --------------------------------------------------------------------------------      40 11F23.93        50.7%   7.5%      --------------------------------------------------------------------------------      41 11F-22.90(1)    43.1%   6.5%      --------------------------------------------------------------------------------      42 11F-22.90(2)    52.1%   6.7%      --------------------------------------------------------------------------------      43 18F-13.28(1)    53.0%   9.4%      --------------------------------------------------------------------------------      44 11F-24.30(3)    62.6%   6.6%      --------------------------------------------------------------------------------      45 11f-0.66(2)     39.1%   6.9%      --------------------------------------------------------------------------------      46 11F-0.66(1)     53.0%   5.8%      --------------------------------------------------------------------------------      47 11F-0.66(3)     51.3%   8.2%      --------------------------------------------------------------------------------      48 11F-24.30(2)    59.2%   6.1%      --------------------------------------------------------------------------------      49 11F-21.42       54.4%   8.9%      --------------------------------------------------------------------------------      50 11F-24.30(1)    49.0%   7.1%      --------------------------------------------------------------------------------      51 11F.26.28       51.8%   4.7%      --------------------------------------------------------------------------------      52 11F-24.30(4)    60.6%   3.4%      --------------------------------------------------------------------------------      53 11F-24.30(6)    51.8%   6.7%      --------------------------------------------------------------------------------         consensus/100%                    ................................................................................         consensus/90%                     ................................................................................         consensus/80%                     ................................................................................         consensus/70%                     ................................................................................                            cov    pid  241          :         .         .         .         .         3         .         . 320   1 18F-16.50(2)   100.0% 100.0%      TSCAV-------TNGPVT---------PCRRRELD----------------------------------IDAI-------       2 18F-15.31       67.1%  21.0%      -------------------------------MVDL----------------------------------LKFV-------       3 18F-16.61       64.3%  25.1%      ---------------------------------MA----------------------------------FNLF-------       4 11F-16.38       66.6%  21.1%      -ICKR-------SYINLS---------NAKKMRFS----------------------------------FSLL-------       5 18F-16.117(1)   59.2%  22.1%      IYCKH-------PVGPA-----------SEEMVHW----------------------------------LSLL-------       6 18F-16.117(2)   61.5%  30.0%      RHVVD-------VETPI-----------LNEMTNL----------------------------------INLV-------       7 18F-16.77       59.5%  20.7%      ---------------MMS---------AVRRV-FA----------------------------------LTFF-------       8 18F-16.102      57.8%  17.9%      ---------------MKI---------KFNNIQIY----------------------------------LSIF-------       9 18F-16.116      48.7%  11.5%      -------------------------------ML-------------SLPFTC-------KPYDRMEASQCCCC-------      10 18F-16.118      65.2%  12.9%      XXXXXXVH----QRSNMF----------HR-------------------------------------FSLVNL-------      11 18F-24.234      47.3%  14.1%      ----------------MK---------NHR-------------------------------------IAITVC-------      12 18F-21-123      47.0%  13.6%      ----------------MQ---------TMT---------------------------------------SHIL-------      13 18F-21.121(1)   45.3%  12.4%      ----------------------------MN--------------------------------------RVGIF-------      14 18F-21.122      46.7%  13.7%      ----------------MR---------QTK--------------------------------------LIGFF-------      15 18F-16.50(1)    45.3%  11.5%      -------------------------------ML-----------------F-----------------------------      16 18F-16.79       41.4%   7.9%      ----------------MS-------TIIRNRMI-----------------G-----------------------------      17 18F-16.153      48.4%   9.0%      ----------------------------MNVL---------------------------------------VC-------      18 18F-12.31       49.0%  12.2%      -------------------------------MTSLL----------ALTLTC-------F--------------------      19 11F-17.59       51.3%  11.6%      --------------------------------------------------------------------------------      20 18F-17.59       51.3%  11.6%      --------------------------------------------------------------------------------      21 18F-18.10       75.6%   9.1%      KIGNAFFN----PGASIN-------VTITNVFTHILPRTTATRTTSFFLMGC-------TPSP----FPYTIC-------      22 18F-18.11       67.1%  10.9%      KLKAKLTN----SSSTSP---------TMNRMG------------VERRVGK-------PDED----KDYEDE-------      23 18F-22.75       66.6%  12.5%      KLKAKFVN----SAIDMS---------SSNTFG------------LLHRVGK-------FDDS----LTFDDK-------      24 18F-10.79(1)    52.7%   5.9%      ENVNQFYYPMSPSFLPTPVR----------DHGRISSRQKT-RPFIRPST---PSDSTDMMANNARFFASFPNL------      25 18F-10.50       47.0%   7.9%      ----HQLYPNNY-----GAR----------AQGGVAERTLEDSGLYQPL----------EPKAESRTFGL----------      26 18F-10.71       46.7%   7.9%      -----QLYPNNY-----GRQ----------APDGMEQRTLEGSNLYERL----------KPKAESRTFGL----------      27 18F-10.68       43.6%   6.9%      ----DVIKKSDG------IQ----------QNKDVIPRN----PG--AL----------LLPQQNRFFV-----------      28 18F-10.69.1     49.0%   5.9%      ----DFIDDKKA--------------------EEILPRN----NI--------------NETPLGRLFN-----------      29 18F-10.69.2     38.2%   7.2%      ----VEILPEIK-----EID----------NQAKIEPE------I--------------QQDNEKVFLG-----------      30 18F-13.28(2)    56.9%   8.7%      ----------------AAAKKNDQSQDWQ---KILKQRSRW----------------------NQKFFLLDNEAQTVH--      31 11F-23.10       55.2%   7.1%      -------EHPRASTDRMMN---TGDRSQQFDDMRVEPRVKNYGHRQQDLTF------GNKPQN--R-FFLTKQ-------      32 11F-2.50        54.7%   8.1%      --GNQEEQDNWVRLLPIVG---YRYKNNPNIEADVKPRIKSS---FLDISS------GKKQGQEARLFYFNSD-------      33 11F-2.51        57.8%   7.4%      KLGFQNEDDKLGKIIPVMA---QQHMTNQNQMANVKPRIKNNGF-FQDLSF------G-NQQQNARLFYLDGS-------      34 11F-13.72(1)    61.2%   8.6%      -----------------TVSK--DIQNDTNEFPDTQSRIRGFGKK-QSS--------LSNNEPAQR-FLLGTSFGNNGYT      35 11F-13.72(2)    70.5%   8.2%      -----------PSFIPTDGGN--HTDDDQNEFPDIQSRIRGFTSN-RFS--------PPNNE--GR-FLIGG-LGNNGLN      36 11F-23.23       43.6%  10.1%      -------QPHFRTV----QN---PKRVHENQLLDVQLRNKLGNSRYWPSFLE-----QLKPQFNPR-IIINLANRT----      37 11F-23.73       54.4%   8.8%      -------QPYSNAI----EN---ANNVDEDQFPDVQSRGKFGVSRYRPWFLQ-----HQTPQYNPR-IVINLASRT----      38 11F-23.98       54.1%   9.5%      -------EPFSNII----QN---SKSVQEDQFPDVELRNKLGNSRYRPSFLQ-----QLKPQFKPR-IIINLASRT----      39 11F-23.53       51.6%   6.5%      -------MPFFRQL----QR---TDPGRKE--ADTKLRNKEL-NRYQP-----------LPPNNAR-FVLNFSSRT----      40 11F23.93        50.7%   7.5%      -------MPFFRQL----PR---DDPDVEEIFGGTQLRNKEM-NRYQP-----------FRQDKAR-LVVNFSSRS----      41 11F-22.90(1)    43.1%   6.5%      ------------------------------DVNNVQARVKGY-EPLPQDLMV-----GQPEIGENR-FFFNSGSSS----      42 11F-22.90(2)    52.1%   6.7%      ------------------------------GVNNIQARVKGY-ETLPQDLFD-----TQDENGENR-FLFNSGSST----      43 18F-13.28(1)    53.0%   9.4%      --------AFQLEEDGAKSDQDWLT------LDSVQSRS--A-NRFRPKLAS----LNQFPPPQQR-RFFGAFNPFFG--      44 11F-24.30(3)    62.6%   6.6%      --------DLITDYVAPDSGKQPINADEPYTFPDTRLRFKGF-SRDQ--------ASNSNDQLDAR-LLFANN--G----      45 11f-0.66(2)     39.1%   6.9%      ---------HRT---A-TDFQD-----S---LEDVQSRTKEL-SLSRPKYQYRPQE---------R-FFVNYA-------      46 11F-0.66(1)     53.0%   5.8%      ---------LNPSYMD-VDYSNDFTEDI---AEDE-FPITES-RNRVPGVRYRPNLPNRVAIPNPR-FFINYY--T----      47 11F-0.66(3)     51.3%   8.2%      ---------LDTSLAT-TEYQDYFDEEE---NSDVQSRFNEF-RQRRPALVKRPQNDER--------LLLSYL--T----      48 11F-24.30(2)    59.2%   6.1%      -------RMFYSGDVESVEK---LLQD-NHNVAQTQP------------RGKT-----SLEEVSPR-FLFGIE-PS----      49 11F-21.42       54.4%   8.9%      --------GFNREKI--------ESHDNLDDFQ-YQSRIRGS---FAQ------------EQVNPR-FFFGLN-------      50 11F-24.30(1)    49.0%   7.1%      --------GFNRGVMAVDESHDVMDDDSHEEFADTQSRINGF-KGFH-------------NHNAGR-FFYAS--------      51 11F.26.28       51.8%   4.7%      --------VLA------TNSQDVGDEQLQ-------VLVGDS-KIGL-------------VDNEGR-FLYSS--------      52 11F-24.30(4)    60.6%   3.4%      --------DYYSGLTSDEDRNNNENEDDQDDYPDIQSRIKWF-NGFNKGFKNK-----GFHHDAGR-FFYSS--------      53 11F-24.30(6)    51.8%   6.7%      --------DLNVDL-SDDGQTNDEVNGPAHQFPDSQQRVNVY-RGFN----------------GGR-FFYSS--------         consensus/100%                    ................................................................................         consensus/90%                     ................................................................................         consensus/80%                     ................................................................................         consensus/70%                     .....................................................................hs.........                            cov    pid  321          .         .         :         .         .         .         .         4 400   1 18F-16.50(2)   100.0% 100.0%      -----LP-NKPIKVETT--AAPEESVSSFIPDIV-S--SSEPRDVLVNGLLPGGTCGPSAGPSVHDN--IARPWLQASKT       2 18F-15.31       67.1%  21.0%      -----LL--LV------------VGFSSVQAAVVIR--EEEIWPNHVVRQVPE-------------F--RAAATVTTVVT       3 18F-16.61       64.3%  25.1%      -----VV--LV------------VALSAVQAAVV--------RPEDVARLLPQ-------------T--RAN-TIISVVT       4 11F-16.38       66.6%  21.1%      ------L--VF------------ITLSSALTQVV--------RQKFV-------------------S--GRTNTLTTFVT       5 18F-16.117(1)   59.2%  22.1%      -----LV--LV------------IALSPVRSAAI--------PVENAAEMVPE-------------N--RAASTFTRIVT       6 18F-16.117(2)   61.5%  30.0%      -----FA--LI------------ITLSSVQSAVV--------REENVADMLAQ-------------N--RAASTVTQVVT       7 18F-16.77       59.5%  20.7%      -----LL-FALLAV-------------EVPARVLDQGDQFAISPSEVSAIV---------EPMVRQARQQSTATVTILKT       8 18F-16.102      57.8%  17.9%      -----L--SILIVD-------------PVPARVVIDPGVDEAG----NGLF---------GGMVQ----GRQITALLTVT       9 18F-16.116      48.7%  11.5%      -----PSKGLIWMF------------IV---LLASGLSL----------------------EIPRQGRQ-----RIESIA      10 18F-16.118      65.2%  12.9%      -----AAVLVVLAF------------SA---VQQVPA-----------------------AAVERDGRTSSA-TITVLST      11 18F-24.234      47.3%  14.1%      -----FIIVLLLAY------------------V--------------------------------DGQR-PT--VTRTTT      12 18F-21-123      47.0%  13.6%      -----FTVLLLLFF------------ST---TT--------------------------------SGQRRRKPQVTKTKT      13 18F-21.121(1)   45.3%  12.4%      -----ATLLLLLSN------------SI-------------------------------------EGDRRVTVVR----M      14 18F-21.122      46.7%  13.7%      -----STLVLLLAF------------SV---TI--------------------------------DGQK-KTVIK----V      15 18F-16.50(1)    45.3%  11.5%      -------KAILIAV------------VC---SMVVAQ--------------S-------DFPVQQETARFLALQVTK---      16 18F-16.79       41.4%   7.9%      -------SILLMAL------------VA---GLTT-V--------------Q-------GWKVQLV------PKTTS---      17 18F-16.153      48.4%   9.0%      -----S---VLL-A------------SV---LLAFGQSEDK------GRTDA-------TGPLRFLVTP-----KTSTAT      18 18F-12.31       49.0%  12.2%      ---------LVLAG------------ES---LAVPVNQTQ---------------------GVNLLSALRMNFPLFSYTT      19 11F-17.59       51.3%  11.6%      -----------MKG------------QS---LM---------------ILLA-------IY--AMFMVSRIDAQRTSTIT      20 18F-17.59       51.3%  11.6%      -----------MKG------------QS---LM---------------ILLA-------IY--AMFMVSRIDAQRTSTIT      21 18F-18.10       75.6%   9.1%      -----PAST----------------------EMDSELLEDD------ERKLI-------HFPVRWSHKK-KKEPIVSTVT      22 18F-18.11       67.1%  10.9%      -----GAGSLELDG------------IE---STIINGTDQE------ERFYN-------KFPLHVFMKKKRPFVSFSTVT      23 18F-22.75       66.6%  12.5%      -----PAVADYDAD------------AA---ELELDGTQEE------ERLFN-------RYPLHVFMKK-KPFVQFSTTT      24 18F-10.79(1)    52.7%   5.9%      -----FFPSMFYS---------------------------------------------------YLSSISTSAYSTSTSY      25 18F-10.50       47.0%   7.9%      --------NNLVS---------------------------------------------------IILSSL-SLSSSSTTY      26 18F-10.71       46.7%   7.9%      --------NYLVS---------------------------------------------------VILSSL-TVSSTSTTY      27 18F-10.68       43.6%   6.9%      ------------N---------------------------------------------------A--NSIYLFTMTTTIK      28 18F-10.69.1     49.0%   5.9%      ------------N---------------------------------------------------ILNPISNLITRTSTIT      29 18F-10.69.2     38.2%   7.2%      ------------N---------------------------------------------------AVLN-FRPFTTTTTFT      30 18F-13.28(2)    56.9%   8.7%      ALRPFNPLEMFSY--------------------------------HIN---Q-------IKPSAPLTR--PIFARRSTVF      31 11F-23.10       55.2%   7.1%      -----Y---HPYK----------------------------------------------------------------TAT      32 11F-2.50        54.7%   8.1%      -----SSINPFLK----------------------------------------------------------------TVT      33 11F-2.51        57.8%   7.4%      -----N---FLSK----------------------------------------------------------------TLT      34 11F-13.72(1)    61.2%   8.6%      GNG--NVRYPFLNTYTTTTTS-------------------------TSTVVT-------LSTSTVMSTSTTSTTSTSTAV      35 11F-13.72(2)    70.5%   8.2%      YNGYYNTDKRFYKTFTTITTTTSTSLSTI-------------FATSISTLVS-------VSTSTTISTSITSTTSTSTVV      36 11F-23.23       43.6%  10.1%      --------NLLNK--------------------------------------------------------------VKTIT      37 11F-23.73       54.4%   8.8%      --------NLLNK--------------------------------------------------------------VKTVT      38 11F-23.98       54.1%   9.5%      --------NLLNK--------------------------------------------------------------VKTIT      39 11F-23.53       51.6%   6.5%      --------HLLDK--------------------------------------------------------------VNTIT      40 11F23.93        50.7%   7.5%      --------YLLNK--------------------------------------------------------------VKTIS      41 11F-22.90(1)    43.1%   6.5%      -----SIGNPLLK----------------------------------------------------------------TVT      42 11F-22.90(2)    52.1%   6.7%      -----SIGNPLLK----------------------------------------------------------------TVT      43 18F-13.28(1)    53.0%   9.4%      -----NNNNLFSSFA-----------------------------------------------------------PQSSTL      44 11F-24.30(3)    62.6%   6.6%      -----IYTNPFLK----------------------------------------------------------------TVT      45 11f-0.66(2)     39.1%   6.9%      ------------K----------------------------------------------------------------TAT      46 11F-0.66(1)     53.0%   5.8%      -----NVASLLTK----------------------------------------------------------------TAT      47 11F-0.66(3)     51.3%   8.2%      --------YFKTT----------------------------------------------------------------TAS      48 11F-24.30(2)    59.2%   6.1%      -----SFTNPFGK----------------------------------------------------------------TAT      49 11F-21.42       54.4%   8.9%      -----PFAGSGSS----------------------------------------------------------------AAV      50 11F-24.30(1)    49.0%   7.1%      -----TINNPFLK----------------------------------------------------------------TAT      51 11F.26.28       51.8%   4.7%      -----VLNNPFIK----------------------------------------------------------------TAT      52 11F-24.30(4)    60.6%   3.4%      -----TINNPFFK----------------------------------------------------------------TAT      53 11F-24.30(6)    51.8%   6.7%      -----TINNPFFK----------------------------------------------------------------TAT         consensus/100%                    ................................................................................         consensus/90%                     ...............................................................................h         consensus/80%                     ..........h..................................................................shs         consensus/70%                     ..........h.t................................................................oho                            cov    pid  401          .         .         .         .         :         .         .         . 480   1 18F-16.50(2)   100.0% 100.0%      --VTA-TVVTTTTKSTFLACATLIEP-----------------LATTACNR--------KKQL-------------WG-V       2 18F-15.31       67.1%  21.0%      --TTA-TDATTITIKKRFDCAKLEAP-----------------LATTVCRR--------KRQY-------------WN-L       3 18F-16.61       64.3%  25.1%      --TTA-VGPTTVTKSTRFVCATLEAP-----------------ISTTACRR--------KRQF-------------WN-A       4 11F-16.38       66.6%  21.1%      --STA-STASTTVVTTRSICASLIEP-----------------VATTPCRR--------KRQF-------------WN-V       5 18F-16.117(1)   59.2%  22.1%      --TTA-EGTTTSVLSTQLVCAILVAP-----------------VATTNCRR--------KKQF-------------WA-V       6 18F-16.117(2)   61.5%  30.0%      --STA-PTASTTVVSSKLICAILVAP-----------------VATTACRR--------KRQF-------------WG-I       7 18F-16.77       59.5%  20.7%      --TTV-SSVLMTTVTTKKTCGILASVNG--TVNPGNNTGFT-NAPTSACNRR-------KRQY-------------WD-E       8 18F-16.102      57.8%  17.9%      --TTV-SSVLTSTVSTSRFCMKLSSSGS---L-----------IPSTQCARNLR-----NGRQ-------------DD-E       9 18F-16.116      48.7%  11.5%      RTVTV-AVTEIAFENKPTLCISLVN-------------------ATRPCVGR--------QAH-------------WSRI      10 18F-16.118      65.2%  12.9%      SLL---PGASTSTLLTKKVCGT---IDG---------------DVSSACRRK--------RQF-------------WIDV      11 18F-24.234      47.3%  14.1%      RTT---TKTDTFYISTYALCASVTTSVS---------------AVVTNCRRR--------RNY-------------WIDV      12 18F-21-123      47.0%  13.6%      EIR---TITETKTKTTSTHCASFITSES---------------EILTTCRRR--------RQY-------------WIDV      13 18F-21.121(1)   45.3%  12.4%      TTE---TTTTTTTISTNTVCFSLNMVGG---------------VPITPCRRR--------RQH-------------WIEE      14 18F-21.122      46.7%  13.7%      STT---TETTTFTKRTSTVCLSLISEGG---------------VGPTPCRRR--------RQF-------------WIEE      15 18F-16.50(1)    45.3%  11.5%      --TTVQLVTVTVDETKKSLCANLVD-------------------VTGACQRR---------KR-------------GMDK      16 18F-16.79       41.4%   7.9%      --VKYVVPVVTSLTTVPSTCYVTVD-------------------VTGDCRRR---------RS-------------AQEK      17 18F-16.153      48.4%   9.0%      VTVKS---TVTAAVGITTSCVKPAA-------------------GLANCRKR---------RN-------------IMEV      18 18F-12.31       49.0%  12.2%      QTSTLTETRVTTQWLPSVVCARLVN-------------------VTGPCRLQPRGQDDNGRIF-------------QMEQ      19 11F-17.59       51.3%  11.6%      RTTTT-VTTVSVTILKSVVCAKLVN-------------------VSGVCRRR--------RGL-------------PIDE      20 18F-17.59       51.3%  11.6%      RTTTT-VTTVSVTILKSVVCAKLVN-------------------VSGVCRRR--------RGL-------------PIDE      21 18F-18.10       75.6%   9.1%      --RTS-IATALITSSTVGLCAQLVN-------------------VTGPCRIH--------RGF-------------RMED      22 18F-18.11       67.1%  10.9%      --QTS-TVTALITSSTVGLCAKLVN-------------------VTGPCRLR--------RGL-------------WEED      23 18F-22.75       66.6%  12.5%      --TTS-TVTALVTLSTVGFCAQLVN-------------------VTGPCRMR--------KGL-------------WVRE      24 18F-10.79(1)    52.7%   5.9%      --LIL-NSTL--TTTLITSCIPISSFSAATL-------------ANVACRRKRHLMDEA--VVAGDDELGVLQ-------      25 18F-10.50       47.0%   7.9%      --TTT-TSTI--TAGTVITCFTKTMFN-----------------ATTACRRKRDALLMISEVLIGDYDD-----------      26 18F-10.71       46.7%   7.9%      --KTT-TSTI--TAGTVITCFTKTMFN-----------------ATTACRRKRDALLMISEVLIGDYDD-----------      27 18F-10.68       43.6%   6.9%      --STV-TSTA--TTAAVVKCIPSTQLS-----------------ATTACARRRRDTEEK--VVIGND-DIVIK-------      28 18F-10.69.1     49.0%   5.9%      --NTF-VSTV--TTATYTHCLPSTQFKIATAADPSATPVVVEVRSTAPCARRRRDVAEF--LAQADSE-IDIH-------      29 18F-10.69.2     38.2%   7.2%      --STS-VSVV--STPTTVKCIPSTQFSIATAAIPAANPPVVEVRATSACARRRRDLEHL--LADADLAKASIE-------      30 18F-13.28(2)    56.9%   8.7%      --FTI-TETT--RKIDVQICIPSTLFTSPSL-------------PTQ-CPTYNRTIRHFKSYVD----------------      31 11F-23.10       55.2%   7.1%      --LTI-TSTC--TAITFKTCVPRANFRPPLP-------------DLVPNCRRKRQAESDLLEDD-LGNQFAIDPXXXXXX      32 11F-2.50        54.7%   8.1%      --LKV-TSTC--TSLSIISCIPAANLPDA-P---------------VPICRRRRDIEMNH-PDE-D--------------      33 11F-2.51        57.8%   7.4%      --VKV-TSTC--TTISIISCIQITNLDPSDP---------------PVPCRRKRSPEVDHTTDD-D--------------      34 11F-13.72(1)    61.2%   8.6%      --ITT-TATL--SLTSVVKCVPQFQVAAGAG-------------P---CGRKKRSTEDFE--------------------      35 11F-13.72(2)    70.5%   8.2%      --VSN-TATL-TLLASLVSCVPQFQVVLGAV-------------A---CPAGRK-KRQIN--------------------      36 11F-23.23       43.6%  10.1%      --FTF-TSSL--TFTSVQSCIPSTDFFPGFN-------------AVA-CRKRRGLFESSD--------------------      37 11F-23.73       54.4%   8.8%      --FTF-TSSV--TFTSVQSCIPSTEFSPGSA-------------GVN-CRKRRGVLESSHS-K-----------------      38 11F-23.98       54.1%   9.5%      --VTF-TSSV--TFTSVQSCIPSTEFFPGLT-------------AVA-CRRKRRELDESPDNK-----------------      39 11F-23.53       51.6%   6.5%      --FTI-TSSV--TFTKRAEI-------------------------------------VESITN-----------------      40 11F23.93        50.7%   7.5%      --FTI-TSSV--TLTKVESCIPSHQFSASFA-------------SVT-CRRKRGGIAELPVTN-----------------      41 11F-22.90(1)    43.1%   6.5%      --FTI-TSTC--TALSITTCVAMSNLSPNPV--------------AC-AGRRRRFIEYSDGQSEDNGTQDDAE----YHY      42 11F-22.90(2)    52.1%   6.7%      --FTI-TSTC--TALSVTTCVVMSNLSPNPV--------------AC-AGRRRRFIEYNNEQGDGV------E----TEY      43 18F-13.28(1)    53.0%   9.4%      --VTI-TASVTLTTAVISTCIPNTQFSVGAA-------------NIV-CARRRRHVAELMDPFD----------------      44 11F-24.30(3)    62.6%   6.6%      --FTY-RTSL--SLTSYVTCVPANQLLNGAG-------------ATY-CRRKRRTINVAADEIA----------------      45 11f-0.66(2)     39.1%   6.9%      --FTV-TSSL--SL--------------------------------T-TCRRKREILDLL-PGP----------------      46 11F-0.66(1)     53.0%   5.8%      --FTV-TSSL--SLTSIQSCIAAVKFLDDAA-------------KTK-ACRRKREILDNV-PHP----------------      47 11F-0.66(3)     51.3%   8.2%      --FTL-TSSV--TLTSVQSCIAAVKFLDDAA-------------KTT-ACRRKRDLLEDS-PSP----------------      48 11F-24.30(2)    59.2%   6.1%      --FTV-SATV--TVSSFVRCIAAAQFQNVAA-------------QGVFCARKRRSLESLE-T-S----------------      49 11F-21.42       54.4%   8.9%      --PTI-MSMV-TTVASIVTCVPAIQFANAAP-------------PPC-VGRKKREIDDVA---S----------------      50 11F-24.30(1)    49.0%   7.1%      --FTI-SSTV-TTVGSIVLCVPSNNLADVPS-------------PTC-AGRKRREVEDSE--------------------      51 11F.26.28       51.8%   4.7%      --FTI-TSTV-TTLGSIILCVPVNNLVAIPS-------------PIC-AGRKKRELEDD---------------------      52 11F-24.30(4)    60.6%   3.4%      --FTI-TSTV-TTLGSVALCVPANNLAANPA-------------PTC-AGRKKREIDDST-TGP----------------      53 11F-24.30(6)    51.8%   6.7%      --FTL-TSTV-TTLASIVICVPANNLAANPA-------------PAC-AGRKRRGMDDNE--------------------         consensus/100%                    ................................................................................         consensus/90%                     .......ss....h.....Ch...........................stt.............................         consensus/80%                     ..ho..sssh..sh.s...Ch...t...................s.s.st+p............................         consensus/70%                     ..hTh.ssos..ohsohhhChs.sp...................sss.CtR+............................                            cov    pid  481          .         5         .         .         .         .         :         . 560   1 18F-16.50(2)   100.0% 100.0%      --FYTLVHDD----TE------QFHPVNPTHV-LKVEPSVLPENYLFFPM-GE-FV------------------Y-PSVQ       2 18F-15.31       67.1%  21.0%      PLFVDPTFVSHDDFQQ------QFQPLKPNKV-IQVAPSRLP---HYPNV-VA-FEP---FAAQQ---QFT---SPYNIQ       3 18F-16.61       64.3%  25.1%      PLFFALGQDDVN----------QFESLN-TQV-LQVEPSALPSMVEYRNR-PV-FRP---YVS-Q---QVN---SPFAIQ       4 11F-16.38       66.6%  21.1%      PLFFALGQQDD---AF------QFQRVNPSQV-LRVEPSVLPEFRY-RPS-SF-VRP---FAGQE---PLS---SPLGLQ       5 18F-16.117(1)   59.2%  22.1%      PVVYTFGQNE----ME------QFDSLNPTRV-SGVETSVLPEFRN-KPA-LP-IN-----SADS---QLS---SNLAIQ       6 18F-16.117(2)   61.5%  30.0%      PIYYTYGQYE----TE------QVVQLDPTQV-LGVESSVQPEYRH-PAS-LP-VN-----SGVS---QFA---SPMAIQ       7 18F-16.77       59.5%  20.7%      PVFVALKEDDNPSLQYF-----R---LSPSQV-LSFEPTMLPQFRNTHNP-SI-AL------------VKE---SPSAIA       8 18F-16.102      57.8%  17.9%      PMLYVLYGENGEDFEFV-----RPDQINPAKI-HSVEATELAQFRDGTPV-EP-AI------------E---------SS       9 18F-16.116      48.7%  11.5%      PPAEL-----AE------------IGVQPSIV-QKVEVTDAPAMSRLNDRDLE-LLK------------LDDRLLLPSLK      10 18F-16.118      65.2%  12.9%      PILIAQDPETAAYIYQQF---------QPSPV-FSVEPTQVVQFRDSFDQPSA-FID-S------SLDEV----TNPRLN      11 18F-24.234      47.3%  14.1%      PVYIALDEDVDDQLTQFF---------HPSST-YEVEPTVGPGFKKDDEDDSG-WLP-DGW-------LA----PSQPIQ      12 18F-21-123      47.0%  13.6%      PVFLAVDEQFDQQLSQYF--------AHPPEK-FQVETTIPAEEGHAHSGWMS-DLF-RSSS---PISSM----SSYSIQ      13 18F-21.121(1)   45.3%  12.4%      PLFLAQQDQVDKDLSQFV--------IEPKET-FQVEPTLLPGAGFNTYGYSG-LNR-GGYG---SRDSL----AKHSIQ      14 18F-21.122      46.7%  13.7%      PMFLEPYNQFDEYYSQFL--------IQPTNV-FQLEKTAAPLLDFRNYPFSG-MDD-EYDS---SAEFF----LPHLPQ      15 18F-16.50(1)    45.3%  11.5%      PIILSFDNDDID---L----------YHPSPV-QQVEATPAVDLDAPG-----------------RVEILESSSE-TPVE      16 18F-16.79       41.4%   7.9%      PTITSMDDMDID---D----------ILPTLP-RL---------DASA-----------------SVESVKPSIDGSRME      17 18F-16.153      48.4%   9.0%      PLILETDDDEEI---------------DPSTP-IEVVITAYPEIRSLK--NAD-SAP-----------VME---VQPSID      18 18F-12.31       49.0%  12.2%      PEVLTFDDEMGE-ADAL-------VQLQPTVT-LKVEPTVMPDNLEANSKM----------------R-LEASWSDPAHK      19 11F-17.59       51.3%  11.6%      PVILTFDDELEDSVDQAFHHVLHPNQFLPTRT-LGVEVTPLVLLP--VQTTVH-SLP-GGYSSPSTVGVVRSSIVGERAR      20 18F-17.59       51.3%  11.6%      PVILTFDDELEDSVDQAFHHVLHPNQFLPTRT-LGVEVTPLVLLP--VQTTVH-SLP-GGYSSPSTVGVVRSSIVGERAR      21 18F-18.10       75.6%   9.1%      PIVLSFDDEMDS-IDSI---------LSPSKT-FSIEATAEPVQGPESEHWVH-A---------RGPRLFAS---DPTIR      22 18F-18.11       67.1%  10.9%      PIVLSFEDDMDS-IDEA---------LSPSKI-FSIETTAMPQDVAEERSG------IDANNNQNRRISVRS---GAVIH      23 18F-22.75       66.6%  12.5%      PIVMSFDDDMDS-IDGA---------LTPSKT-VGIETTARPGEIADVIQERE-TSTTVPLNSTSQRSSVRS---GPLIQ      24 18F-10.79(1)    52.7%   5.9%      --------------GP--------NYLSNEE--EQWMDSSLDN---SL-----------------------GRTVDPEIL      25 18F-10.50       47.0%   7.9%      --------------QD--------EP-LGDD--NEWDGELEPL---PREK----------------RD---VDLAR-LIA      26 18F-10.71       46.7%   7.9%      --------------QD--------EP-LGDE--NEWDSELEPL---PREK----------------RD---VDPAR-LIA      27 18F-10.68       43.6%   6.9%      --------------PD--------PP-KPLE-----TSVMSDD---IPEA----------------AP----SVALPDIS      28 18F-10.69.1     49.0%   5.9%      --------------PA--------EP-LPIE-----TSAVVDT---PSEI----------------RE---IQPAQTELL      29 18F-10.69.2     38.2%   7.2%      --------------PA--------QP-IALE-----VSAVEPL---PVAD----------------AKE--ASFTEPEIE      30 18F-13.28(2)    56.9%   8.7%      -----L-------LEG--------ENIRPSST-SPVMPTVLNIQS--------------------------QQPPVYSIE      31 11F-23.10       55.2%   7.1%      XXXXXXDLLEDDLDNQ--------FAIDPSQV-QLVMPTEQPMKVSFIHD---------------------GREITREIM      32 11F-2.50        54.7%   8.1%      -------------RMQ--------FSVNPSNV-QPVVPTAVPWPAVPRDS---------------------AQISSVDVT      33 11F-2.51        57.8%   7.4%      -------------ESQ--------FPINPSKV-ETVTPTMEPLSILPRDL---------------------LQTPSVEMT      34 11F-13.72(1)    61.2%   8.6%      -------N-----EPQ--------FAINPSAT-LKVTPTALPSESTLLREIRELGLP---------S-D--ERQVIGSLR      35 11F-13.72(2)    70.5%   8.2%      -------D-----SEQ--------FIIAPSET-LELTTTALPSLDVEEQS-TESDLP---------VED--EQKNVNALA      36 11F-23.23       43.6%  10.1%      -----------LGTSQ--------FTITPSDV-QPMEPTVLSWLDPSVENQQSSG-----------NDP----TVNASVV      37 11F-23.73       54.4%   8.8%      -----------LETSQ--------FAIAPSDV-QRVEQTVLTSQDPTAGN-GPLA-----------NDA----AANHGVV      38 11F-23.98       54.1%   9.5%      ----------LATLQQ--------FAITPSNV-QSVEPTVLSPVDPTAVK---PG-----------NDA----IVNQGIV      39 11F-23.53       51.6%   6.5%      -----------WKYIQ--------LAIKPTYV-QPVEPTVISALNLLTGNA-----------------------ELPLIS      40 11F23.93        50.7%   7.5%      -----------WKDIQ--------LATKPTNV-QPVEPTIVSALNSPTGSA-----------------------ELPQIS      41 11F-22.90(1)    43.1%   6.5%      PEYRDFDDPHTNIATQ--------FPISASEV-RIVMPTTGPSTGPARNSRD---------------------LSFGIAS      42 11F-22.90(2)    52.1%   6.7%      PE-----HLLSNIEAQ--------YPIVPSPI-KIVMPTAEPSTGLARNSHD---------------------SPFGISS      43 18F-13.28(1)    53.0%   9.4%      -------DIEPNSFLQ--------TQVSPTQV-LKVEPTAIPLANIERERRQ---------------------ADFQGIV      44 11F-24.30(3)    62.6%   6.6%      -------E-----GNQ--------FLIAPSET-QKLATTAISSPSRNLD----------------------------QLS      45 11f-0.66(2)     39.1%   6.9%      -------E-----STQ--------FPIIPSKIKEHVMPTALPSLGLNRE-NRQVS-----------YDQ----S------      46 11F-0.66(1)     53.0%   5.8%      -------E-----DLQ--------FAIVPSQT-QQVTPTAVPSLELNGD-QVDSF-----------D-------------      47 11F-0.66(3)     51.3%   8.2%      -------A-----DLQ--------FAIIPSET-QQVIPTTVPSLEVTRE-HRQMS-----------SDQ----AFQHDIA      48 11F-24.30(2)    59.2%   6.1%      -------E-----SQA--------DAIAPSVP-QLLATASFPSIDLLKEGRSQDG-----------RTN--QQTQQNVIV      49 11F-21.42       54.4%   8.9%      -------E-----HIQ--------FPIVPTAT-LKLTPTHLVRENRQL------S-----------TDQ----SIRDILH      50 11F-24.30(1)    49.0%   7.1%      -------------HLQ--------FPIVPSET-VKLTSTALPYLHTGS-----AI-----------NET--I---SDDLV      51 11F.26.28       51.8%   4.7%      --------------NQ--------FPIVPSET-LSLMPTAVPPADKSV-----AL-----------GTP--VQQNSRGLF      52 11F-24.30(4)    60.6%   3.4%      -------D-----DNQ--------FAIEPSET-LELMPTALPSDVGLARESRVMM-----------HTQ--KKTSPQRLI      53 11F-24.30(6)    51.8%   6.7%      -------------THQ--------FPITPSET-LTLTPTAVPSLELEA---RQLF-----------ADE---EKNEHELI         consensus/100%                    ................................................................................         consensus/90%                     ............................ss.....h.ss..s....................................h.         consensus/80%                     ...............t..........h.Poph...l.soh.s....................................h.         consensus/70%                     ...............p..........ltPops..tltsTshs.....h.............................tlt                            cov    pid  561          .         .         .         6         .         .         .         . 640   1 18F-16.50(2)   100.0% 100.0%      SSLNGGPIE---------SPTRIAD-PFL------FST---------------------------FISSLFSNLFPGTSS       2 18F-15.31       67.1%  21.0%      SSLDKTSIQ---------PALRVAD-PLFGAFYAFSSLLSNIF--------------------AGMLRPQSTPAVLVTST       3 18F-16.61       64.3%  25.1%      SSLSS--KV---------QAVRVAD-PFFGAAR--FSAFSSLF--------------------SGIISSILTPAVTSTST       4 11F-16.38       66.6%  21.1%      PSFDRMQPV----------SFRVAD-PFFGAVK--FSALSAIY--------------------ANILNSLLTPATTVTRT       5 18F-16.117(1)   59.2%  22.1%      SSIDGIDDVQ--------PMIRVAN-PLF------FRPVASII--------------------LNALSSMRNPPITVT--       6 18F-16.117(2)   61.5%  30.0%      PSMDGTGQLVP-----VYSMFRVAD-PFF------FRPFSSLI--------------------SSFLSSLLTPPVTVTST       7 18F-16.77       59.5%  20.7%      PSFGKSH-----------ENVRVAS-PIF------GGGINSLI--------------------SSIVSGILITTTTSTSI       8 18F-16.102      57.8%  17.9%      LETDDPD-----------NGVRVAQ-PIY------FAALASLL--------------------SNIFPSALQSTTTSTIT       9 18F-16.116      48.7%  11.5%      ETLE--------------ENGRQQS---SGFLRRFTDR-----------FS-------------NALGL---ARTVVQTV      10 18F-16.118      65.2%  12.9%      GALL----SPV-----NTVRTSL--------------------L----AIA------------SNVINSFLTPTVTVTSV      11 18F-24.234      47.3%  14.1%      PSLM----TPV-----TDGGFTE---ERAPEP----IGFAVIAE----AIN------------NALIAVGLADPTTFVTE      12 18F-21-123      47.0%  13.6%      PSI------QE-----NDF-KPI----FFGEL----DDMARRM-------------------------FGRRRKVTFVTN      13 18F-21.121(1)   45.3%  12.4%      SSLF----VPV-----SSEHQRL----YFGSP----LFLRRIWA----YLA------------NDL--YRRDDTKRTVTF      14 18F-21.122      46.7%  13.7%      SSLW----MPS-----TDFRPRI----YFKNG----DTFARGG------FR------------RAG--NRKTSKKTTITF      15 18F-16.50(1)    45.3%  11.5%      VDIV----G---------LQSECYG-RRFGLVSALASGVASVLP----EVSSDL-----ASSLTSVLNLTLRFK-----T      16 18F-16.79       41.4%   7.9%      ENIE----S---------------K-RGWTFTARPLDGFSKVNR----WIS----------DNRPAFGLTYVDF-----E      17 18F-16.153      48.4%   9.0%      NTAGMAEVQ-I-----HDMDSICKARLANPFF---------------------------GAGLAQVFGITVLFST---TV      18 18F-12.31       49.0%  12.2%      GEGIDGQSS----------------------P---------------------------GLT--DAI---RAFFNVVQIE      19 11F-17.59       51.3%  11.6%      NSGRNKLMSSQ-----SDEEGRPEQKIYFAQL---------------------------GQLI-SSIANQLRPPVLTTTT      20 18F-17.59       51.3%  11.6%      NSGRNKLMSSQ-----SDEEGRPEQKIYFAQL---------------------------GQLI-SSIANQLRPPVLTTTT      21 18F-18.10       75.6%   9.1%      SSAS----AEQ-----SDEADRQRL-GFHNLL---------------------------GLK--LALKKRIKYITIVTTV      22 18F-18.11       67.1%  10.9%      SSKDDGESK---------DDDDGNE-GRFGFF---------------------------GLK--KKFKKKIKFITVITTT      23 18F-22.75       66.6%  12.5%      SSKEDAELKQE-----SDDDEQVEQ-GRFGYF---------------------------GLK--KTFK-KIKFVTVVTTT      24 18F-10.79(1)    52.7%   5.9%      SSKETEV------------TGNHLPQ-YLLRQAR-------------------------------------SARYTITST      25 18F-10.50       47.0%   7.9%      SSKTESDLHVNDVAP--KQTESSVDGYFRQNRA--------------------------------------IVTSTLTSY      26 18F-10.71       46.7%   7.9%      SSKTESDLHVNDAAP--KQTESSVDGFFRKNRS--------------------------------------IVTSTLTSY      27 18F-10.68       43.6%   6.9%      SSQR--E-------L--ETGEFSD---HQQRAAF-------------------------------------TTTVSVTSL      28 18F-10.69.1     49.0%   5.9%      SSKS--DELVENPEI--RQGSNS----------N-------------------------------------AATKLVTVI      29 18F-10.69.2     38.2%   7.2%      SS----QLMNLSPEV--VQDDPSKEDRGLLNLSL-------------------------------------TTTISVTAV      30 18F-13.28(2)    56.9%   8.7%      SSKDEDLNEERDLGYYEMEELTRRDRAMFGWPT--------------------------------------------TRT      31 11F-23.10       55.2%   7.1%      SSKEEEGRGSR---LVDASEMQMRNKKRFLYFG--------------------------------------PSALVAGTT      32 11F-2.50        54.7%   8.1%      SSKEVEINNSKLSGISTQFEEPDREARF-LW----------------------------------------KHQFT-STT      33 11F-2.51        57.8%   7.4%      SSKEEAVKNDESNSLVTYMAKQQREGKAFKFNI--------------------------------------QNFLTYTTV      34 11F-13.72(1)    61.2%   8.6%      SSKTEVYLDEAP----EEEQKTIREKRFFLANKNYFFT----------WTSATTSTSTLVVSVTSVIQSITTLTAFATTT      35 11F-13.72(2)    70.5%   8.2%      SSKDETHLND---------GDNLRDKRFLNRYQFSTSTSTSLTTSTTTSTSVSTSTSIITVPTTSVTVSTSILTSLTTTT      36 11F-23.23       43.6%  10.1%      SSKDDGTLDEWQSVTLKPLGHYSRDKRLYLH-------------------------------------------LVMTKT      37 11F-23.73       54.4%   8.8%      SSKDEDTLDELSNVTLKPVG-YLRDKRFFFH-------------------------------------------LVTTTT      38 11F-23.98       54.1%   9.5%      SSKDEDTLEELSNVTLKPVG-YLRDKRFFFH-------------------------------------------LVTTTT      39 11F-23.53       51.6%   6.5%      SSKDEDLLNEKQNSQEMPLSKQSRMKRLFFH-------------------------------------------LVTTTT      40 11F23.93        50.7%   7.5%      SSKDEDLLNEKQSSQVIPPSQQARMKRLLFH-------------------------------------------FVATTT      41 11F-22.90(1)    43.1%   6.5%      SMEDDGASN----EL-VRVNTQIRGKRFFQQ---------------------------------------LWNGFA----      42 11F-22.90(2)    52.1%   6.7%      SMKDDVEAN----GF-VSKDRKLRGKRFFN-----------------------------------------WNAFATGVT      43 18F-13.28(1)    53.0%   9.4%      SSKEDVESLANDSK--DQAMINQRQNRAFT---V-----------------------------------------TFSTT      44 11F-24.30(3)    62.6%   6.6%      SSKDDAALE-E--LS-AIDQSQIRRQRLL---------------------------------------FLNRNQFVISST      45 11f-0.66(2)     39.1%   6.9%      ---DYLSSG-EISGS-ISPEKHLRGKRFFKFYS------------------------------------------VASTT      46 11F-0.66(1)     53.0%   5.8%      ---DNVSVG-ELSGS-ISAAQNLRERRFF-KYS------------------------------------------VTSTT      47 11F-0.66(3)     51.3%   8.2%      SSKGLASSA-DLSGS-VSWLKQMRDPRFLNTYS------------------------------------------LASTT      48 11F-24.30(2)    59.2%   6.1%      SSKDETENGPEST----TSYANLKNKRFIF--------------------------------------FHNKNAYVTSTT      49 11F-21.42       54.4%   8.9%      STKDDITNH-E--VS-NTDQTNMRDRRFFLSNGILSNGLLGN------------------------GLFGSSSAMGAASV      50 11F-24.30(1)    49.0%   7.1%      SSKDEWMGTVEMPDE-ENKDNNSREKRFFG-GGL-----------------------------------------AASTT      51 11F.26.28       51.8%   4.7%      SSKEETTDSSESSLE-DDANDNPRAKRFFG-GGV-----------------------------------------AASTT      52 11F-24.30(4)    60.6%   3.4%      SSKDEVLVSPFEKPS-EEDENSLREKRIFGGTGF-----------------------------------------AASTT      53 11F-24.30(6)    51.8%   6.7%      SSKEVV--NKFV----PVEAEKPREKRFFG-GGI-----------------------------------------AASTT         consensus/100%                    ................................................................................         consensus/90%                     sph........................h....................................................         consensus/80%                     soht...............t.p.....hh..............................................hhssh         consensus/70%                     uShp....t..........t.p.tt..hh.............................................hshoos                            cov    pid  641          :         .         .         .         .         7         .         . 720   1 18F-16.50(2)   100.0% 100.0%      ATVYTS-TVTSTEFTATSTY----------------------TLSAVSCIPVD--LITCPPTPDATVVTEDPITEDPVT-       2 18F-15.31       67.1%  21.0%      KTVYTT-TDTVTSTTSTATY----------------------TLKGAHCLPPG--VALCPGSATSSITPEATSTTTAATS       3 18F-16.61       64.3%  25.1%      KTVYTT-TTTTTSFTSTAIY----------------------TIKNAQCVPSG--VKLCPVSSTTTSTTPSTTTVTGEPT       4 11F-16.38       66.6%  21.1%      TTVFPA-TITNTLFTETVTL----------------------TLNQAACVPAG--LTICPAAPTTTTTPSTTTTPSTTTT       5 18F-16.117(1)   59.2%  22.1%      --------------------------------------------------------------------------------       6 18F-16.117(2)   61.5%  30.0%      STVFTQ-TITRTSFTATATF----------------------TLTSLPCVPAD--VSLCPAAP-----------------       7 18F-16.77       59.5%  20.7%      ITMYTS-TSVSTSFTATTSF----------------------FVVG-ACYPAG--LTIC---------------------       8 18F-16.102      57.8%  17.9%      STVYNGGTTFITVYTSTNSF----------------------FLLG-SCYPSF--PPLC---------------------       9 18F-16.116      48.7%  11.5%      TETRTM-TTTYTESSGYNHF----------------------YISD--CIPPTLYYPICKKYTDDAE-------------      10 18F-16.118      65.2%  12.9%      VNGYSS-TTTTTIFTTTQTY----------------------TVAG--CIPEGVEFSAC---------------------      11 18F-24.234      47.3%  14.1%      IETNTK-TTTKTTTTSTKTF----------------------YLSG--CKPSPFPFTQCK--------------------      12 18F-21-123      47.0%  13.6%      TFTTTS--TTTKTSTTTATF----------------------FLTG--CVPSPFSYSFCS--------------------      13 18F-21.121(1)   45.3%  12.4%      TRTKIA--TEREMSTKTNTI----------------------GISG--CTPSPLPYDVC---------------------      14 18F-21.122      46.7%  13.7%      TRTRLV--TETEMSTKTNTI----------------------GISG--CTPSPLFYDICP--------------------      15 18F-16.50(1)    45.3%  11.5%      TLTATV-TVTNTVTDGSKAF----------------------VVSG--CTPSPFLYQTC---------------------      16 18F-16.79       41.4%   7.9%      TVTLTR-TTYSTVTDGQKTF----------------------VVSG--CLPAGFLYTTC---------------------      17 18F-16.153      48.4%   9.0%      TATQQV-TSTTTTVAKRNTI----------------------ELAG--CLPNPLPFSLC---------------------      18 18F-12.31       49.0%  12.2%      RTTSTV-QSTTFLPFTTKTI----------------------FIMK--CTPLPFTVSICSRGPKGPRRD-----------      19 11F-17.59       51.3%  11.6%      ITRTNT-RTTTTTTFSTASF----------------------FVMS--CTPSPFPFSVCPAKRFHAD-------------      20 18F-17.59       51.3%  11.6%      ITRTNT-RTTTTTTFSTASF----------------------FVMS--CTPSPFPFSVCPAKRFHAD-------------      21 18F-18.10       75.6%   9.1%      FVTITS-TSVDYITVSTKTF----------------------FVQI--CTPSPFPFDVCVRSRR----------------      22 18F-18.11       67.1%  10.9%      VVTSTS-TSTYYVTISTKTF----------------------FIQL--CTPSPFPFNICNGRKK-RQAQLEEMNRASNQN      23 18F-22.75       66.6%  12.5%      AVTATS-TSTYYVTISTKTF----------------------FIQI--CTPSPFPFNVCSVGTKKRHIEFEP--------      24 18F-10.79(1)    52.7%   5.9%      FTSYSF---FTVNST--KTAFL---------------------GSSLFCMPGG--FKTC---------------------      25 18F-10.50       47.0%   7.9%      TVSYST------SSRIVTNIAS---------------------ESALSCLPSG--FTLC---------------------      26 18F-10.71       46.7%   7.9%      TVSYST------SSRIVTNIAS---------------------ASALSCLPSG--FTLC---------------------      27 18F-10.68       43.6%   6.9%      TTTFVF---VATSITSTTSLGI---------------------SAGLLCLPSG--FTICA--------------------      28 18F-10.69.1     49.0%   5.9%      ATSFTF---VAKTLTSTATLAA---------------------AEGLLCRPPG--YAMKLALIFLTCFVSLSYQQRY---      29 18F-10.69.2     38.2%   7.2%      TTVFSF---AGATVKNTASIAA---------------------DGALQCLPSG--FLVC---------------------      30 18F-13.28(2)    56.9%   8.7%      VTVVAT---QTSIVTRAVSPMH---------------PPIGSPTGFLICVPPG--LVVCV--------------------      31 11F-23.10       55.2%   7.1%      ITSWSV---VSTIITSTFVP-----------------------AVPFRCLPPG--FTICP--------------------      32 11F-2.50        54.7%   8.1%      TTSWSV---VSSTLTQTFVP-----------------------AADFDCLPPG--YVVC---------------------      33 11F-2.51        57.8%   7.4%      TILGSI---VTSTLTQTLVP-----------------------VEPLACLPPG--YVLCPL-------------------      34 11F-13.72(1)    61.2%   8.6%      VSSFLF---ANSTITQTINLIT-PVPVAQCVPAAAVPP--APAINCLSCLPNG--YVVCA--------------------      35 11F-13.72(2)    70.5%   8.2%      MTSFVF---VNQTITQTANLITNPVPTAQCAGDADGDGVVTPPSAHLSHVYQQ--VWSCALLLANRFSKALQQLIDY---      36 11F-23.23       43.6%  10.1%      VISYTS---LSTTLTKTVNLLN-P----------F-----F-GAGQLVCLPQG--YAVCPNNATSFPR------------      37 11F-23.73       54.4%   8.8%      AISYTF---LSTTVTKTVNLLS-P----------M-----PAPIGQLVCLPQG--YSVCPYLGTLPPR------------      38 11F-23.98       54.1%   9.5%      AISYTF---LSTTVTKTVNLLS-P----------M-----PAPIGQLVCLPQG--YSVCPYLGTLPPR------------      39 11F-23.53       51.6%   6.5%      VVSYTF---FSATITKTVNLLS-V----------V-----EQGPGYLICRLEG--FLVCSSSSTINT-------------      40 11F23.93        50.7%   7.5%      VVSYTF---FSATSTKTVSLLS-V----------A-----DQGPGFLICRPEG--YSVCS--------------------      41 11F-22.90(1)    43.1%   6.5%      --------------------------------------------------------------------------------      42 11F-22.90(2)    52.1%   6.7%      ITTWSV---ISSTLTSTLAIP--------------------GVSTVLPCLPAG--YGST---------------------      43 18F-13.28(1)    53.0%   9.4%      TTSYFF---TSTVVRKTLNLA---------------------ASTALSCVPVD---------------------------      44 11F-24.30(3)    62.6%   6.6%      VTSFSF---SNATVTVTKNLFN-ANQAAQCLAAPD-----MDIPQCIACLPPG--FIVCNAADINEMPFGIFNISHSHLN      45 11f-0.66(2)     39.1%   6.9%      ITTYSI---TSTTSTKTVAVA---------------------GDGE----------------------------------      46 11F-0.66(1)     53.0%   5.8%      VTTFSI---LSTTVTKTVAVA---------------------VTDLYK---KR--TSVCERNHSTSV-----------EG      47 11F-0.66(3)     51.3%   8.2%      LTSYMF---TSTTVTKTVAIA---------------------ADGLVLCLPTG--YIVC---------------------      48 11F-24.30(2)    59.2%   6.1%      VTSLNF---ITSISTTTANIR--------------------VAGALLLCVPIG--YRFITGVAAADQ-----------QC      49 11F-21.42       54.4%   8.9%      VTSYAF---VATTLIQTVNLVT-SL-TTQC--GTG-----TGTFPCADCLPAG--YIVCTP-------------------      50 11F-24.30(1)    49.0%   7.1%      MTQYLF---VGTTLTSTVILDP-T------------------GKNVAVCLPAG--Y------------------------      51 11F.26.28       51.8%   4.7%      LTSYSF---VGATVTSTVLLDP-T------------------GANLAACLPAG--YVVCF--MTLTH-----------IP      52 11F-24.30(4)    60.6%   3.4%      LTSYSF---VGATVTNTVLLDP-T------------------AGGLAACLPSG--YLEVVIVVSIAI-----------VL      53 11F-24.30(6)    51.8%   6.7%      VVSYSF---IGATITSTVLLDP-T------------------AGGLAACLPAG--YVVCA--------------------         consensus/100%                    ................................................................................         consensus/90%                     h.............h.h...............................Ch.....h..h.....................         consensus/80%                     hhshs....htt..ptThsh........................t...ChP.s..h.hC.....................         consensus/70%                     hsshsh...hssshspThsh.......................hs...ChPss..a.hC.....................                            cov    pid  721          .         .         :         .         .         .         .         8 800   1 18F-16.50(2)   100.0% 100.0%      ---------DGWILIKTIRRR------SK---------------------------------------------------       2 18F-15.31       67.1%  21.0%      TSTSTAATTTTEGTTSAATDSTTTGSTSTTTTDATTTT-S-------TDS-TTTDSTSTTS---------TTESSTTTTP       3 18F-16.61       64.3%  25.1%      TPTTT--TTTGETTSTTTTDATTTTTGATTTTEATTTT---------TEA-TTTTTDATT-----------TTTDATTTT       4 11F-16.38       66.6%  21.1%      PSTTTTPST-------TTTP-------STTTTPSTTTA-P-------SNT-TTL--------------------------       5 18F-16.117(1)   59.2%  22.1%      --------------------------------------------------------------------------------       6 18F-16.117(2)   61.5%  30.0%      --------------------------------------------------------------------------------       7 18F-16.77       59.5%  20.7%      --------------------------------------------------------------------------------       8 18F-16.102      57.8%  17.9%      --------------------------------------------------------------------------------       9 18F-16.116      48.7%  11.5%      --------------------------------------------------------------------------------      10 18F-16.118      65.2%  12.9%      --------------------------------------------------------------------------------      11 18F-24.234      47.3%  14.1%      --------------------------------------------------------------------------------      12 18F-21-123      47.0%  13.6%      --------------------------------------------------------------------------------      13 18F-21.121(1)   45.3%  12.4%      --------------------------------------------------------------------------------      14 18F-21.122      46.7%  13.7%      --------------------------------------------------------------------------------      15 18F-16.50(1)    45.3%  11.5%      --------------------------------------------------------------------------------      16 18F-16.79       41.4%   7.9%      --------------------------------------------------------------------------------      17 18F-16.153      48.4%   9.0%      --------------------------------------------------------------------------------      18 18F-12.31       49.0%  12.2%      --------------------------------------------------------------------------------      19 11F-17.59       51.3%  11.6%      --------------------------------------------------------------------------------      20 18F-17.59       51.3%  11.6%      --------------------------------------------------------------------------------      21 18F-18.10       75.6%   9.1%      --------------------------------------------------------------------------------      22 18F-18.11       67.1%  10.9%      S-------------------------------------------------------------------------------      23 18F-22.75       66.6%  12.5%      --------------------------------------------------------------------------------      24 18F-10.79(1)    52.7%   5.9%      --------------------------------------------------------------------------------      25 18F-10.50       47.0%   7.9%      --------------------------------------------------------------------------------      26 18F-10.71       46.7%   7.9%      --------------------------------------------------------------------------------      27 18F-10.68       43.6%   6.9%      --------------------------------------------------------------------------------      28 18F-10.69.1     49.0%   5.9%      --------------------------------------------------------------------------------      29 18F-10.69.2     38.2%   7.2%      --------------------------------------------------------------------------------      30 18F-13.28(2)    56.9%   8.7%      --------------------------------------------------------------------------------      31 11F-23.10       55.2%   7.1%      --------------------------------------------------------------------------------      32 11F-2.50        54.7%   8.1%      --------------------------------------------------------------------------------      33 11F-2.51        57.8%   7.4%      --------------------------------------------------------------------------------      34 11F-13.72(1)    61.2%   8.6%      --------------------------------------------------------------------------------      35 11F-13.72(2)    70.5%   8.2%      --------------------------------------------------------------------------------      36 11F-23.23       43.6%  10.1%      --------------------------------------------------------------------------------      37 11F-23.73       54.4%   8.8%      --------------------------------------------------------------------------------      38 11F-23.98       54.1%   9.5%      --------------------------------------------------------------------------------      39 11F-23.53       51.6%   6.5%      --------------------------------------------------------------------------------      40 11F23.93        50.7%   7.5%      --------------------------------------------------------------------------------      41 11F-22.90(1)    43.1%   6.5%      --------------------------------------------------------------------------------      42 11F-22.90(2)    52.1%   6.7%      --------------------------------------------------------------------------------      43 18F-13.28(1)    53.0%   9.4%      --------------------------------------------------------------------------------      44 11F-24.30(3)    62.6%   6.6%      AD-GTKENGATDIGHCTLRDVLVGA-------------------------------------------------GPRSTF      45 11f-0.66(2)     39.1%   6.9%      --------------------------------------------------------------------------------      46 11F-0.66(1)     53.0%   5.8%      KT-SSS---------SVLQSAK----------------------------------------------------------      47 11F-0.66(3)     51.3%   8.2%      --------------------------------------------------------------------------------      48 11F-24.30(2)    59.2%   6.1%      RG-DHR-----------FRQTVAQVK------------------------------------------------------      49 11F-21.42       54.4%   8.9%      --------------------------------------------------------------------------------      50 11F-24.30(1)    49.0%   7.1%      --------------------------------------------------------------------------------      51 11F.26.28       51.8%   4.7%      AR-LTTPISE----------------IHS-----------------------EKDRYITTK------------------T      52 11F-24.30(4)    60.6%   3.4%      AS-ETAEKEGVEIGISIMAQLIVLVDINRESVAATCELFPRVFALNKSNKINTKRAYINKIVFQCKYCHLTIATRATSRF      53 11F-24.30(6)    51.8%   6.7%      --------------------------------------------------------------------------------         consensus/100%                    ................................................................................         consensus/90%                     ................................................................................         consensus/80%                     ................................................................................         consensus/70%                     ................................................................................                            cov    pid  801          .         .         .         .         :         .         .         . 880   1 18F-16.50(2)   100.0% 100.0%      --------------------------------------------------------------------------------       2 18F-15.31       67.1%  21.0%      ASGR----------------------------------------------------------------------------       3 18F-16.61       64.3%  25.1%      TSGRR---------------------------------------------------------------------------       4 11F-16.38       66.6%  21.1%      --------------------------------------------------------------------------------       5 18F-16.117(1)   59.2%  22.1%      --------------------------------------------------------------------------------       6 18F-16.117(2)   61.5%  30.0%      --------------------------------------------------------------------------------       7 18F-16.77       59.5%  20.7%      --------------------------------------------------------------------------------       8 18F-16.102      57.8%  17.9%      --------------------------------------------------------------------------------       9 18F-16.116      48.7%  11.5%      --------------------------------------------------------------------------------      10 18F-16.118      65.2%  12.9%      --------------------------------------------------------------------------------      11 18F-24.234      47.3%  14.1%      --------------------------------------------------------------------------------      12 18F-21-123      47.0%  13.6%      --------------------------------------------------------------------------------      13 18F-21.121(1)   45.3%  12.4%      --------------------------------------------------------------------------------      14 18F-21.122      46.7%  13.7%      --------------------------------------------------------------------------------      15 18F-16.50(1)    45.3%  11.5%      --------------------------------------------------------------------------------      16 18F-16.79       41.4%   7.9%      --------------------------------------------------------------------------------      17 18F-16.153      48.4%   9.0%      --------------------------------------------------------------------------------      18 18F-12.31       49.0%  12.2%      --------------------------------------------------------------------------------      19 11F-17.59       51.3%  11.6%      --------------------------------------------------------------------------------      20 18F-17.59       51.3%  11.6%      --------------------------------------------------------------------------------      21 18F-18.10       75.6%   9.1%      --------------------------------------------------------------------------------      22 18F-18.11       67.1%  10.9%      --------------------------------------------------------------------------------      23 18F-22.75       66.6%  12.5%      --------------------------------------------------------------------------------      24 18F-10.79(1)    52.7%   5.9%      --------------------------------------------------------------------------------      25 18F-10.50       47.0%   7.9%      --------------------------------------------------------------------------------      26 18F-10.71       46.7%   7.9%      --------------------------------------------------------------------------------      27 18F-10.68       43.6%   6.9%      --------------------------------------------------------------------------------      28 18F-10.69.1     49.0%   5.9%      -------------------------------------VWPMPYAPRAHL-------------------------------      29 18F-10.69.2     38.2%   7.2%      --------------------------------------------------------------------------------      30 18F-13.28(2)    56.9%   8.7%      --------------------------------------------------------------------------------      31 11F-23.10       55.2%   7.1%      --------------------------------------------------------------------------------      32 11F-2.50        54.7%   8.1%      --------------------------------------------------------------------------------      33 11F-2.51        57.8%   7.4%      --------------------------------------------------------------------------------      34 11F-13.72(1)    61.2%   8.6%      --------------------------------------------------------------------------------      35 11F-13.72(2)    70.5%   8.2%      --------------------------------------------------------------------------------      36 11F-23.23       43.6%  10.1%      --------------------------------------------------------------------------------      37 11F-23.73       54.4%   8.8%      --------------------------------------------------------------------------------      38 11F-23.98       54.1%   9.5%      --------------------------------------------------------------------------------      39 11F-23.53       51.6%   6.5%      --------------------------------------------------------------------------------      40 11F23.93        50.7%   7.5%      --------------------------------------------------------------------------------      41 11F-22.90(1)    43.1%   6.5%      --------------------------------------------------------------------------------      42 11F-22.90(2)    52.1%   6.7%      --------------------------------------------------------------------------------      43 18F-13.28(1)    53.0%   9.4%      --------------------------------------------------------------------------------      44 11F-24.30(3)    62.6%   6.6%      STVEQIEYT-QKFHLLLK--------------------------------------------------------------      45 11f-0.66(2)     39.1%   6.9%      --------------------------------------------------------------------------------      46 11F-0.66(1)     53.0%   5.8%      --------------------------------------------------------------------------------      47 11F-0.66(3)     51.3%   8.2%      --------------------------------------------------------------------------------      48 11F-24.30(2)    59.2%   6.1%      --------------------------------------------------------------------------------      49 11F-21.42       54.4%   8.9%      --------------------------------------------------------------------------------      50 11F-24.30(1)    49.0%   7.1%      --------------------------------------------------------------------------------      51 11F.26.28       51.8%   4.7%      PLWRLSKYNAMKLTLILLVCFVGVTY-------QQGYAWSP-FHRH-SPR--LYLSNNYHENDRQLP---------AKSA      52 11F-24.30(4)    60.6%   3.4%      SSHSCLKF--MKFVLLFVTSVLSVSTHQYLHKTPAGLVWLSPYMSKQKPVLHKYQQVLYEPVDPDMPYFRYSKPLHPAVT      53 11F-24.30(6)    51.8%   6.7%      --------------------------------------------------------------------------------         consensus/100%                    ................................................................................         consensus/90%                     ................................................................................         consensus/80%                     ................................................................................         consensus/70%                     ................................................................................                            cov    pid  881          .         9         .         .         .         .         :         . 960   1 18F-16.50(2)   100.0% 100.0%      --------------------------------------------------------------------------------       2 18F-15.31       67.1%  21.0%      --------------------------------------------------------------------------------       3 18F-16.61       64.3%  25.1%      --------------------------------------------------------------------------------       4 11F-16.38       66.6%  21.1%      --------------------------------------------------------------------------------       5 18F-16.117(1)   59.2%  22.1%      --------------------------------------------------------------------------------       6 18F-16.117(2)   61.5%  30.0%      --------------------------------------------------------------------------------       7 18F-16.77       59.5%  20.7%      --------------------------------------------------------------------------------       8 18F-16.102      57.8%  17.9%      --------------------------------------------------------------------------------       9 18F-16.116      48.7%  11.5%      --------------------------------------------------------------------------------      10 18F-16.118      65.2%  12.9%      --------------------------------------------------------------------------------      11 18F-24.234      47.3%  14.1%      --------------------------------------------------------------------------------      12 18F-21-123      47.0%  13.6%      --------------------------------------------------------------------------------      13 18F-21.121(1)   45.3%  12.4%      --------------------------------------------------------------------------------      14 18F-21.122      46.7%  13.7%      --------------------------------------------------------------------------------      15 18F-16.50(1)    45.3%  11.5%      --------------------------------------------------------------------------------      16 18F-16.79       41.4%   7.9%      --------------------------------------------------------------------------------      17 18F-16.153      48.4%   9.0%      --------------------------------------------------------------------------------      18 18F-12.31       49.0%  12.2%      --------------------------------------------------------------------------------      19 11F-17.59       51.3%  11.6%      --------------------------------------------------------------------------------      20 18F-17.59       51.3%  11.6%      --------------------------------------------------------------------------------      21 18F-18.10       75.6%   9.1%      --------------------------------------------------------------------------------      22 18F-18.11       67.1%  10.9%      --------------------------------------------------------------------------------      23 18F-22.75       66.6%  12.5%      --------------------------------------------------------------------------------      24 18F-10.79(1)    52.7%   5.9%      --------------------------------------------------------------------------------      25 18F-10.50       47.0%   7.9%      --------------------------------------------------------------------------------      26 18F-10.71       46.7%   7.9%      --------------------------------------------------------------------------------      27 18F-10.68       43.6%   6.9%      --------------------------------------------------------------------------------      28 18F-10.69.1     49.0%   5.9%      --------------------------------------------------------------------------------      29 18F-10.69.2     38.2%   7.2%      --------------------------------------------------------------------------------      30 18F-13.28(2)    56.9%   8.7%      --------------------------------------------------------------------------------      31 11F-23.10       55.2%   7.1%      --------------------------------------------------------------------------------      32 11F-2.50        54.7%   8.1%      --------------------------------------------------------------------------------      33 11F-2.51        57.8%   7.4%      --------------------------------------------------------------------------------      34 11F-13.72(1)    61.2%   8.6%      --------------------------------------------------------------------------------      35 11F-13.72(2)    70.5%   8.2%      --------------------------------------------------------------------------------      36 11F-23.23       43.6%  10.1%      --------------------------------------------------------------------------------      37 11F-23.73       54.4%   8.8%      --------------------------------------------------------------------------------      38 11F-23.98       54.1%   9.5%      --------------------------------------------------------------------------------      39 11F-23.53       51.6%   6.5%      --------------------------------------------------------------------------------      40 11F23.93        50.7%   7.5%      --------------------------------------------------------------------------------      41 11F-22.90(1)    43.1%   6.5%      --------------------------------------------------------------------------------      42 11F-22.90(2)    52.1%   6.7%      --------------------------------------------------------------------------------      43 18F-13.28(1)    53.0%   9.4%      --------------------------------------------------------------------------------      44 11F-24.30(3)    62.6%   6.6%      --------------------------------------------------------------------------------      45 11f-0.66(2)     39.1%   6.9%      --------------------------------------------------------------------------------      46 11F-0.66(1)     53.0%   5.8%      --------------------------------------------------------------------------------      47 11F-0.66(3)     51.3%   8.2%      --------------------------------------------------------------------------------      48 11F-24.30(2)    59.2%   6.1%      --------------------------------------------------------------------------------      49 11F-21.42       54.4%   8.9%      --------------------------------------------------------------------------------      50 11F-24.30(1)    49.0%   7.1%      --------------------------------------------------------------------------------      51 11F.26.28       51.8%   4.7%      YDFELADSLATYDD---DEIASSLSDVQARIPFGS-ARKAQKKFFVTSVFPNSLFPFNFRPVTVTTLTTTALSVITSTAV      52 11F-24.30(4)    60.6%   3.4%      YHQQ-KDEQFEYDDAHHDDINEVFVDSQSRSKGSQLQEKSNPRFFFGTA-----------TTIRNPFIKTATFTLFTTIS      53 11F-24.30(6)    51.8%   6.7%      --------------------------------------------------------------------------------         consensus/100%                    ................................................................................         consensus/90%                     ................................................................................         consensus/80%                     ................................................................................         consensus/70%                     ................................................................................                            cov    pid  961          .         .         .         0         .         .         .         . 1040  1 18F-16.50(2)   100.0% 100.0%      --------------------------------------------------------------------------------       2 18F-15.31       67.1%  21.0%      --------------------------------------------------------------------------------       3 18F-16.61       64.3%  25.1%      --------------------------------------------------------------------------------       4 11F-16.38       66.6%  21.1%      --------------------------------------------------------------------------------       5 18F-16.117(1)   59.2%  22.1%      --------------------------------------------------------------------------------       6 18F-16.117(2)   61.5%  30.0%      --------------------------------------------------------------------------------       7 18F-16.77       59.5%  20.7%      --------------------------------------------------------------------------------       8 18F-16.102      57.8%  17.9%      --------------------------------------------------------------------------------       9 18F-16.116      48.7%  11.5%      --------------------------------------------------------------------------------      10 18F-16.118      65.2%  12.9%      --------------------------------------------------------------------------------      11 18F-24.234      47.3%  14.1%      --------------------------------------------------------------------------------      12 18F-21-123      47.0%  13.6%      --------------------------------------------------------------------------------      13 18F-21.121(1)   45.3%  12.4%      --------------------------------------------------------------------------------      14 18F-21.122      46.7%  13.7%      --------------------------------------------------------------------------------      15 18F-16.50(1)    45.3%  11.5%      --------------------------------------------------------------------------------      16 18F-16.79       41.4%   7.9%      --------------------------------------------------------------------------------      17 18F-16.153      48.4%   9.0%      --------------------------------------------------------------------------------      18 18F-12.31       49.0%  12.2%      --------------------------------------------------------------------------------      19 11F-17.59       51.3%  11.6%      --------------------------------------------------------------------------------      20 18F-17.59       51.3%  11.6%      --------------------------------------------------------------------------------      21 18F-18.10       75.6%   9.1%      --------------------------------------------------------------------------------      22 18F-18.11       67.1%  10.9%      --------------------------------------------------------------------------------      23 18F-22.75       66.6%  12.5%      --------------------------------------------------------------------------------      24 18F-10.79(1)    52.7%   5.9%      --------------------------------------------------------------------------------      25 18F-10.50       47.0%   7.9%      --------------------------------------------------------------------------------      26 18F-10.71       46.7%   7.9%      --------------------------------------------------------------------------------      27 18F-10.68       43.6%   6.9%      --------------------------------------------------------------------------------      28 18F-10.69.1     49.0%   5.9%      --------------------------------------------------------------------------------      29 18F-10.69.2     38.2%   7.2%      --------------------------------------------------------------------------------      30 18F-13.28(2)    56.9%   8.7%      --------------------------------------------------------------------------------      31 11F-23.10       55.2%   7.1%      --------------------------------------------------------------------------------      32 11F-2.50        54.7%   8.1%      --------------------------------------------------------------------------------      33 11F-2.51        57.8%   7.4%      --------------------------------------------------------------------------------      34 11F-13.72(1)    61.2%   8.6%      --------------------------------------------------------------------------------      35 11F-13.72(2)    70.5%   8.2%      --------------------------------------------------------------------------------      36 11F-23.23       43.6%  10.1%      --------------------------------------------------------------------------------      37 11F-23.73       54.4%   8.8%      --------------------------------------------------------------------------------      38 11F-23.98       54.1%   9.5%      --------------------------------------------------------------------------------      39 11F-23.53       51.6%   6.5%      --------------------------------------------------------------------------------      40 11F23.93        50.7%   7.5%      --------------------------------------------------------------------------------      41 11F-22.90(1)    43.1%   6.5%      --------------------------------------------------------------------------------      42 11F-22.90(2)    52.1%   6.7%      --------------------------------------------------------------------------------      43 18F-13.28(1)    53.0%   9.4%      --------------------------------------------------------------------------------      44 11F-24.30(3)    62.6%   6.6%      --------------------------------------------------------------------------------      45 11f-0.66(2)     39.1%   6.9%      --------------------------------------------------------------------------------      46 11F-0.66(1)     53.0%   5.8%      --------------------------------------------------------------------------------      47 11F-0.66(3)     51.3%   8.2%      --------------------------------------------------------------------------------      48 11F-24.30(2)    59.2%   6.1%      --------------------------------------------------------------------------------      49 11F-21.42       54.4%   8.9%      --------------------------------------------------------------------------------      50 11F-24.30(1)    49.0%   7.1%      --------------------------------------------------------------------------------      51 11F.26.28       51.8%   4.7%      LATVQSCIPAAQFLTQQAAGGPMVFTTACGRRRRGIDQPDRQIEKQFDDSISPTAVL----P-----LVTSSVAPSFDGY      52 11F-24.30(4)    60.6%   3.4%      LSSVVSCIVAGDFVDAAAQA------KPCRRKRDLVHQSDQTNEQYFISPSEPQKFTATAVPYPSDLMRDKRQLPIFQGT      53 11F-24.30(6)    51.8%   6.7%      --------------------------------------------------------------------------------         consensus/100%                    ................................................................................         consensus/90%                     ................................................................................         consensus/80%                     ................................................................................         consensus/70%                     ................................................................................                            cov    pid 1041          :         .         .         .         .         1         .        ] 1119  1 18F-16.50(2)   100.0% 100.0%      -------------------------------------------------------------------------------       2 18F-15.31       67.1%  21.0%      -------------------------------------------------------------------------------       3 18F-16.61       64.3%  25.1%      -------------------------------------------------------------------------------       4 11F-16.38       66.6%  21.1%      -------------------------------------------------------------------------------       5 18F-16.117(1)   59.2%  22.1%      -------------------------------------------------------------------------------       6 18F-16.117(2)   61.5%  30.0%      -------------------------------------------------------------------------------       7 18F-16.77       59.5%  20.7%      -------------------------------------------------------------------------------       8 18F-16.102      57.8%  17.9%      -------------------------------------------------------------------------------       9 18F-16.116      48.7%  11.5%      -------------------------------------------------------------------------------      10 18F-16.118      65.2%  12.9%      -------------------------------------------------------------------------------      11 18F-24.234      47.3%  14.1%      -------------------------------------------------------------------------------      12 18F-21-123      47.0%  13.6%      -------------------------------------------------------------------------------      13 18F-21.121(1)   45.3%  12.4%      -------------------------------------------------------------------------------      14 18F-21.122      46.7%  13.7%      -------------------------------------------------------------------------------      15 18F-16.50(1)    45.3%  11.5%      -------------------------------------------------------------------------------      16 18F-16.79       41.4%   7.9%      -------------------------------------------------------------------------------      17 18F-16.153      48.4%   9.0%      -------------------------------------------------------------------------------      18 18F-12.31       49.0%  12.2%      -------------------------------------------------------------------------------      19 11F-17.59       51.3%  11.6%      -------------------------------------------------------------------------------      20 18F-17.59       51.3%  11.6%      -------------------------------------------------------------------------------      21 18F-18.10       75.6%   9.1%      -------------------------------------------------------------------------------      22 18F-18.11       67.1%  10.9%      -------------------------------------------------------------------------------      23 18F-22.75       66.6%  12.5%      -------------------------------------------------------------------------------      24 18F-10.79(1)    52.7%   5.9%      -------------------------------------------------------------------------------      25 18F-10.50       47.0%   7.9%      -------------------------------------------------------------------------------      26 18F-10.71       46.7%   7.9%      -------------------------------------------------------------------------------      27 18F-10.68       43.6%   6.9%      -------------------------------------------------------------------------------      28 18F-10.69.1     49.0%   5.9%      -------------------------------------------------------------------------------      29 18F-10.69.2     38.2%   7.2%      -------------------------------------------------------------------------------      30 18F-13.28(2)    56.9%   8.7%      -------------------------------------------------------------------------------      31 11F-23.10       55.2%   7.1%      -------------------------------------------------------------------------------      32 11F-2.50        54.7%   8.1%      -------------------------------------------------------------------------------      33 11F-2.51        57.8%   7.4%      -------------------------------------------------------------------------------      34 11F-13.72(1)    61.2%   8.6%      -------------------------------------------------------------------------------      35 11F-13.72(2)    70.5%   8.2%      -------------------------------------------------------------------------------      36 11F-23.23       43.6%  10.1%      -------------------------------------------------------------------------------      37 11F-23.73       54.4%   8.8%      -------------------------------------------------------------------------------      38 11F-23.98       54.1%   9.5%      -------------------------------------------------------------------------------      39 11F-23.53       51.6%   6.5%      -------------------------------------------------------------------------------      40 11F23.93        50.7%   7.5%      -------------------------------------------------------------------------------      41 11F-22.90(1)    43.1%   6.5%      -------------------------------------------------------------------------------      42 11F-22.90(2)    52.1%   6.7%      -------------------------------------------------------------------------------      43 18F-13.28(1)    53.0%   9.4%      -------------------------------------------------------------------------------      44 11F-24.30(3)    62.6%   6.6%      -------------------------------------------------------------------------------      45 11f-0.66(2)     39.1%   6.9%      -------------------------------------------------------------------------------      46 11F-0.66(1)     53.0%   5.8%      -------------------------------------------------------------------------------      47 11F-0.66(3)     51.3%   8.2%      -------------------------------------------------------------------------------      48 11F-24.30(2)    59.2%   6.1%      -------------------------------------------------------------------------------      49 11F-21.42       54.4%   8.9%      -------------------------------------------------------------------------------      50 11F-24.30(1)    49.0%   7.1%      -------------------------------------------------------------------------------      51 11F.26.28       51.8%   4.7%      DDVQPELRSSQTNLEEDQRQIFRVDAGNARQRRGL--SLVLTVTLTSTSYSFSTTTLKKTVNLSGTGQLSCLPAGFAVC      52 11F-24.30(4)    60.6%   3.4%      SLWNKDIIES--TKEENDVSYFWP-AKNRKEQRFLFENSHYVASTTVTSYNFVSTTVTKTVDVAIDVGLNCVPTGYV--      53 11F-24.30(6)    51.8%   6.7%      -------------------------------------------------------------------------------         consensus/100%                    ...............................................................................         consensus/90%                     ...............................................................................         consensus/80%                     ...............................................................................         consensus/70%                     ............................................................................... ``` |

MView 1.63, Copyright © 1997-2018 Nigel P. Brown
