# Supplementary figures and images for: QTL study reveals candidate genes underlying host resistance in a Red Queen model system
Source: PLoS Genet. 2023 Feb 2;19(2):e1010570. doi: 10.1371/journal.pgen.1010570 (PMC9894429; doi:10.1371/journal.pgen.1010570)

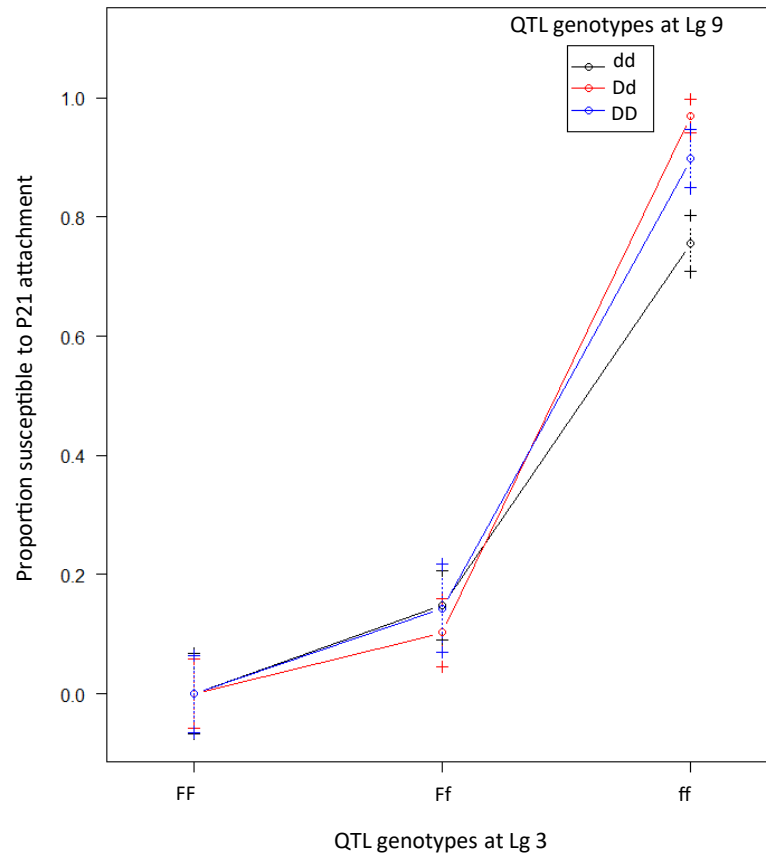

|                            | df | LOD    | % variance | P-value (Chi <sup>2</sup> ) |
|----------------------------|----|--------|------------|-----------------------------|
| Lg 3 @ 157.0 (F locus QTL) | 6  | 65.115 | 56.844     | < 2e-16                     |
| Lg 9 @ 105.3 (D locus QTL) | 6  | 4.481  | 2.513      | 0.00213                     |
| Lg 3 x Lg 9                | 4  | 2.478  | 1.371      | 0.02231                     |

Supplement: S2 Fig — Effect plot showing mean susceptibility (± 1 SE) as a function of genotype at two putative QTL explaining host variation in hindgut attachment of Pasteuria ramosa genotype P21. The x axis shows variation at the QTL detected on linkage group 3 (lg3) at position 157.0 (near F locus), and different colors represent the genotypes at the QTL on lg9 at position 105.3 (near D locus). The Xinb3 QTL parent is known to be susceptible to both parasite genotypes, with genotype ff at lg3 and DD at lg9. The Iinb1 QTL parent is known to be resistant to both parasite genotypes, with genotype FF at lg3 and dd at lg9. Table shows estimated support of each QTL after dropping one term at a time from a binary multiple-QTL model with both QTL (lg3 @ 157.0 and lg9 @ 105.3) and an interaction term (lg3 x lg9) between them. For each model term, we give the degrees of freedom (df), the log10 likelihood ratio (LOD) comparing the full model to reduced models, the estimated percent of phenotypic variance explained by the term, and a p-value that is based on the LOD score and assumes a χ2 distribution of LOD x (2ln10). Note that p-values are pointwise, meaning they do not account for the search over the whole genome. We therefore consider them with caution. (PDF) [file pgen.1010570.s003.pdf]

x*F*i*F*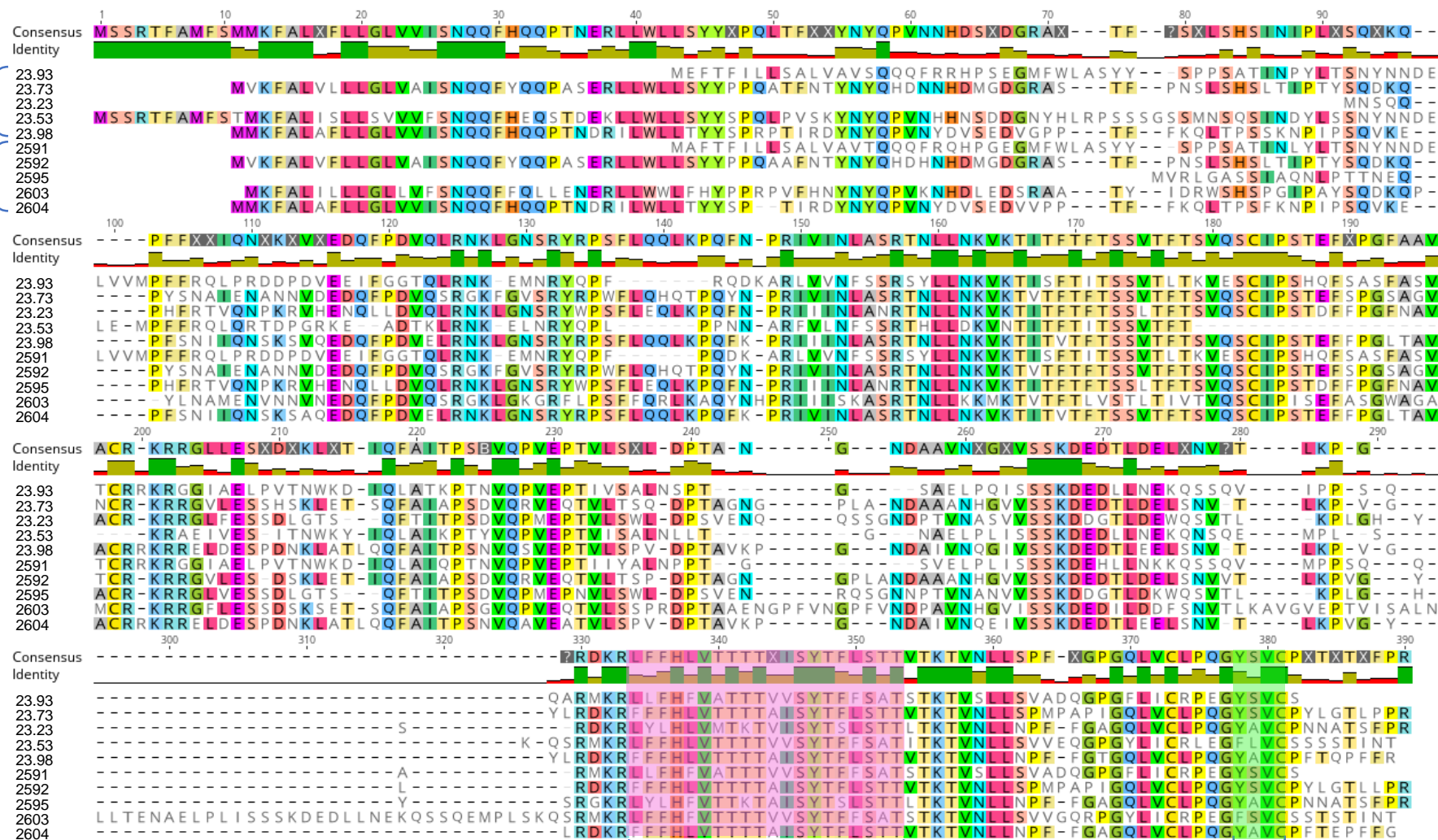

Supplement: S4 Fig — Predicted sequences of five Cladoceran-specific (type I) paralogs from each of xF and iF were aligned using MAFFT in Geneious. The predicted transmembrane domain and STAT motif are indicated (pink and green, respectively). (PDF) [file pgen.1010570.s005.pdf]

A

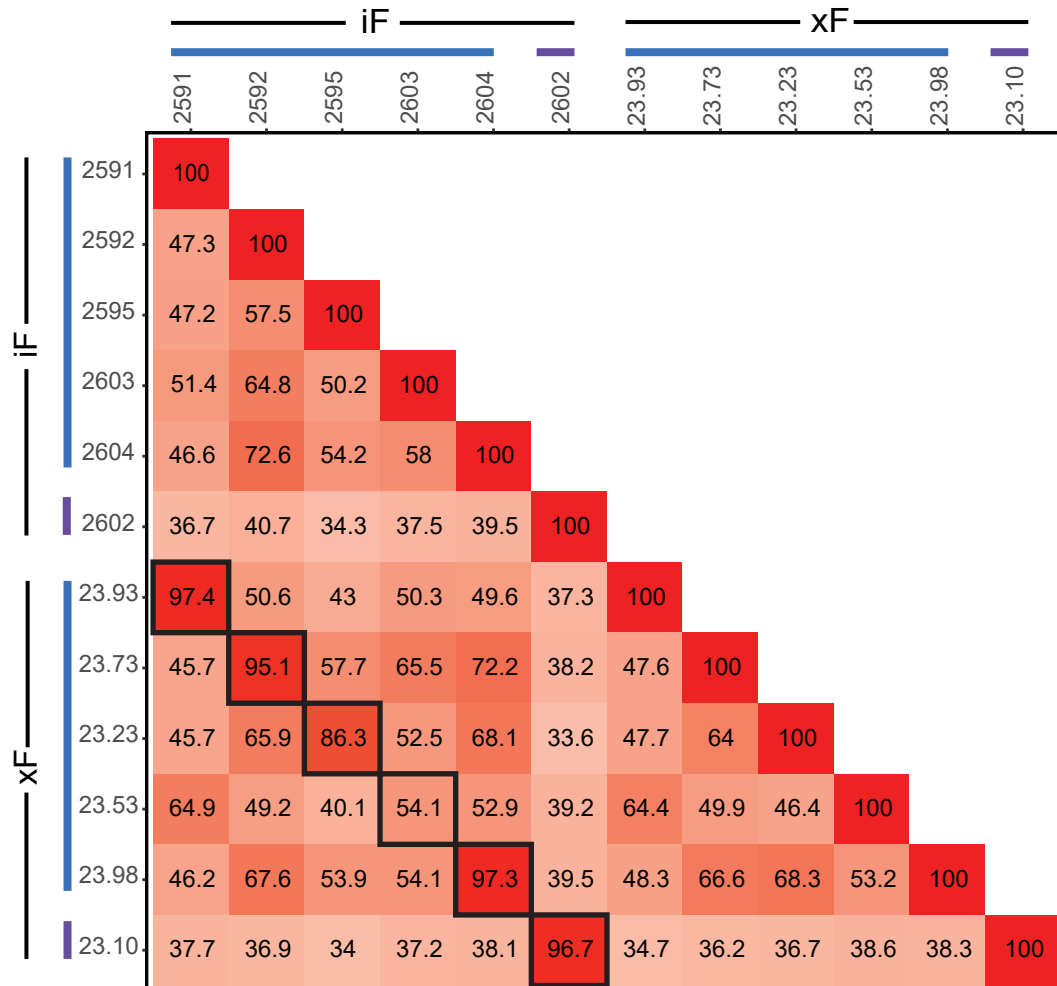

B

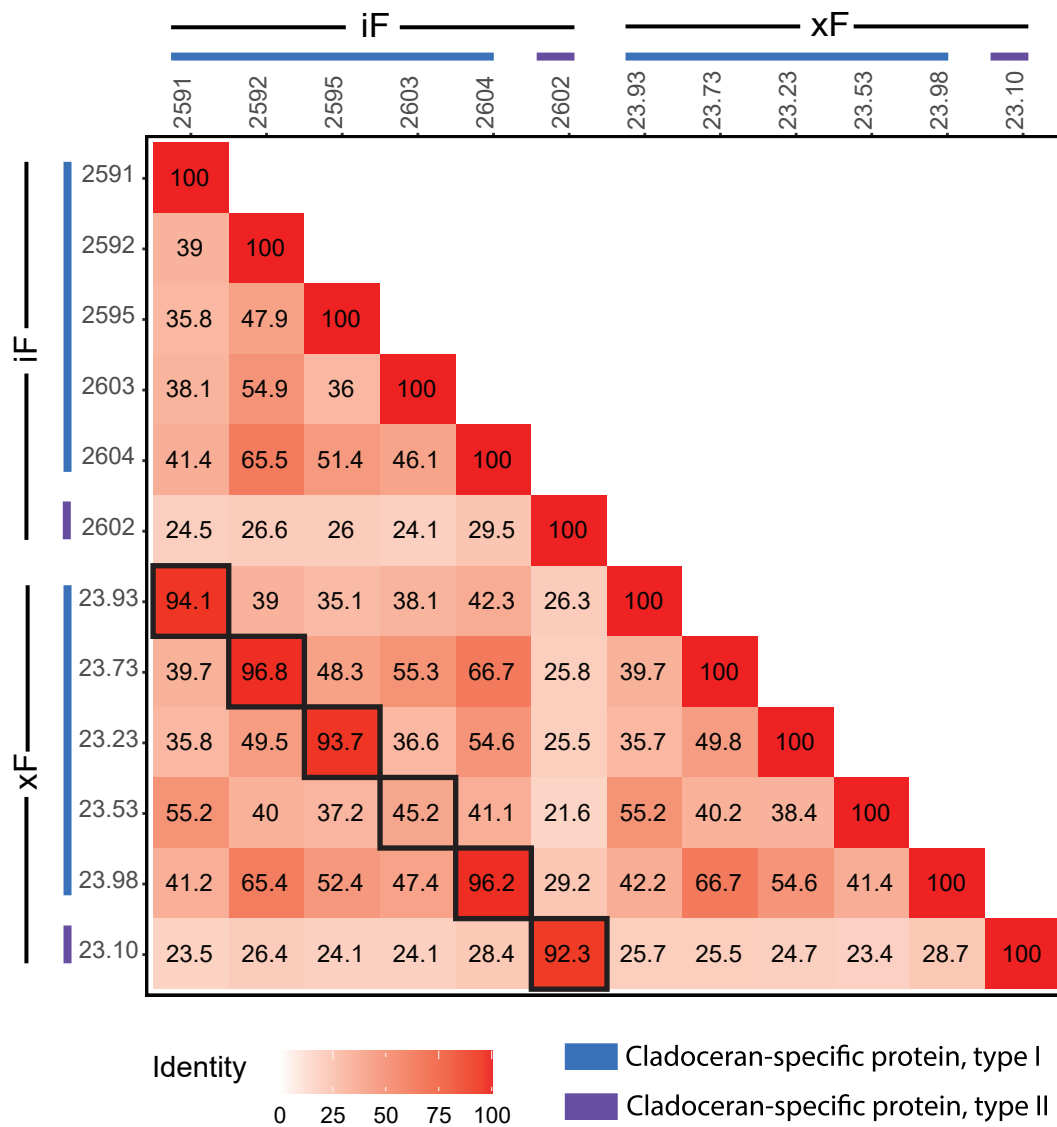

Supplement: S5 Fig — Heatmaps comparing percent identities of six Cladoceran-specific genes, including five type I (blue) and one type II (purple) from each of xF and iF. Percent identities were calculated from pairwise alignments of full mRNA sequences (A) and predicted protein sequences (B). Bold black outline indicates positional homologs (compared in Table 2). (PDF) [file pgen.1010570.s006.pdf]

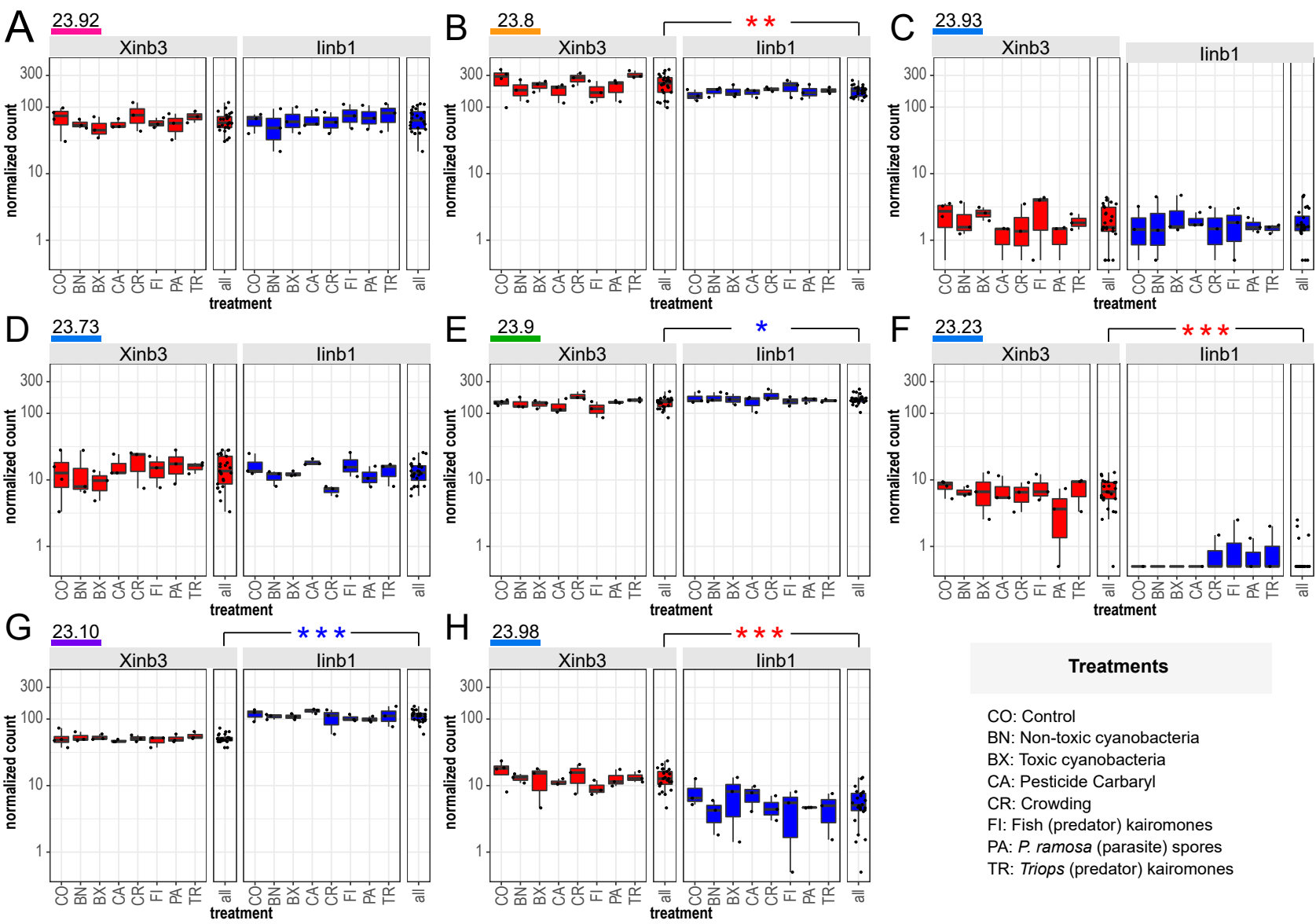

Supplement: S6 Fig — Boxplots showing gene expression data collected across multiple stressful conditions for a previous study [30], with raw RNA-seq reads from QTL parent clones Xinb3 and Iinb1 mapped to the genome-based transcriptome from clone Xinb3. Gene names are underlined with colors corresponding to functional annotation (see Fig 1). Y-axes from each plot show normalized read counts with a pseudocount of 0.5 added to allow for log-scale plotting. Each plotted point represents a Daphnia magna individual from the respective clone. Box edges indicate first and third quartiles, central line indicates median, and whiskers extend to 1.5 x interquartile range. Asterisks indicate genes that show significant differential expression (non-zero logarithmic fold change in mean expression across all treatments combined) between QTL parent clones Xinb3 (n = 25) and Iinb1(n = 24), with clone Xinb3 as reference after correcting for multiple tests: Benjamini-Hochberg-adjusted p-value < 0.05 (*), < 0.01 (**), < 0.001 (***). Asterisks are colored according to the parent clone which shows higher expression (red = susceptible parent Xinb3; blue = resistant parent Iinb1). Wald test statistics and Benjamini-Hochberg-adjusted p-values are as follows: A) z = 0.425, p = 0.498; B) z = -2.78, p = 9.96E-03; C) z = -0.488, p = 0.690; D) z = -0.898, p = 0.442; E) z = 2.59, p = 0.0167; F) z = -8.89, p = 4.00E-18; G) z = 13.23, p = 8.51e-39; H) z = -6.23, p = 1.80E-09. (PDF) [file pgen.1010570.s007.pdf]

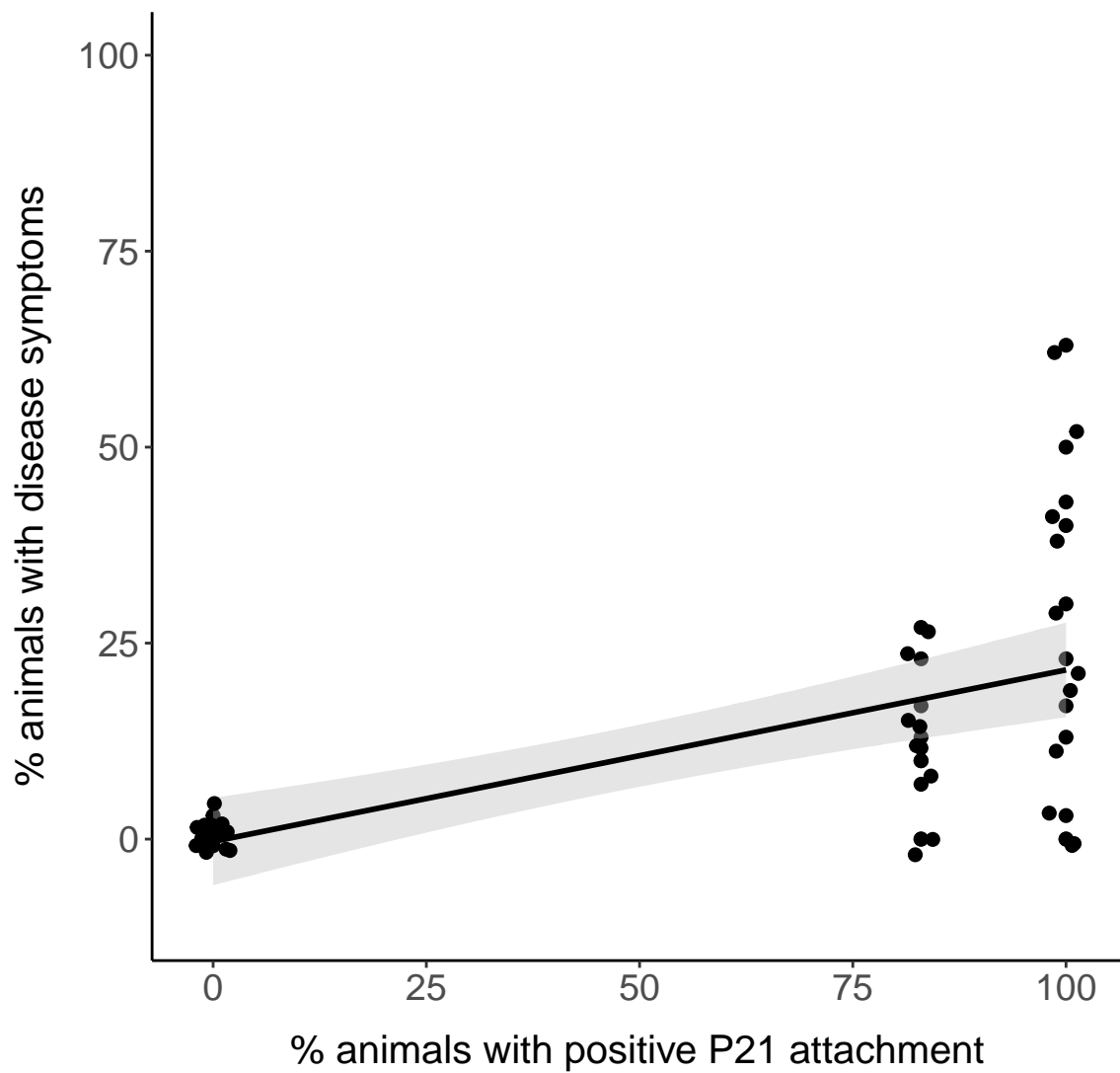

Supplement: S8 Fig — Scatterplot showing percent of individuals with positive attachment to the hindgut and percent of exposed individuals showing disease symptoms five weeks after infection. Each point represents one of 40 Daphnia magna clones from the QTL F2 panel, tested against spores from the Pasteuria ramosa genotype P21. The linear regression line is shown in black, with gray shading indicating the 95% confidence interval. Positive attachment was strongly correlated with subsequent infection (Spearman’s rho = 0.76, P < 0.001, n = 40). (PDF) [file pgen.1010570.s009.pdf]

A

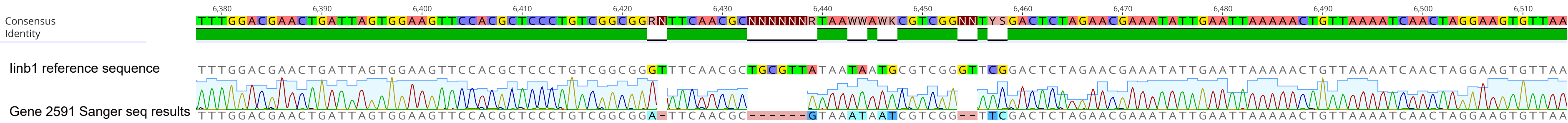

B

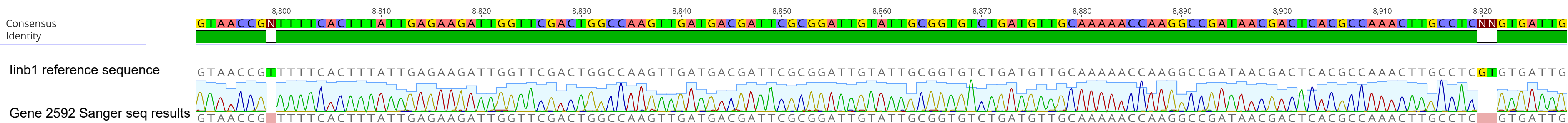

C

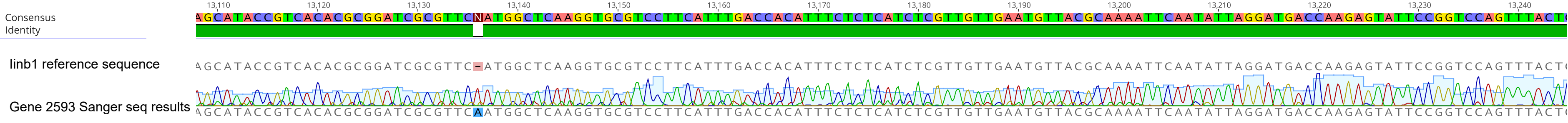

Supplement: S9 Fig — Nucleotide sequence alignments showing the iF haplotype extracted from the Iinb1 reference genome (upper sequence in each panel) aligned to Sanger-sequencing data (lower sequence in each panel), which were used to correct the sequences of iF genes 2591 (A), 2592 (B), and 2593 (C) for comparison with xF homologs (summarized in Table 2). Sanger sequencing results include associated chromatograms, colored by the nucleotide base called at the given peak. Annotations above the aligned sequences indicate the consensus sequence (colored by base identity and numbered according to the position in the iF haplotype) and the identity score of the alignments (green indicates 100% identity and gaps indicate polymorphisms). Raw sequencing data can be accessed at the GenBank accession numbers provided at the end of the manuscript. Figure was created using Geneious Prime software. (PDF) [file pgen.1010570.s010.pdf]
