# Supplementary material for: QTL study reveals candidate genes underlying host resistance in a Red Queen model system
Source: PLoS Genet. 2023 Feb 2;19(2):e1010570. doi: 10.1371/journal.pgen.1010570 (PMC9894429; doi:10.1371/journal.pgen.1010570)
Supplement: S3 Fig — Extra-locus duplications from the F-locus region and their mapping locations in the respective QTL parent genome (for full BLAST results see S2 File). Duplicated segments (black) are separated and labeled according to the contig to which they map (leading zeroes in contig names are omitted). Colored arrows indicate annotated genes in the F-locus region (see Fig 1 for color code). A) Extra-locus duplications from xF and their mapping locations in the Xinb3 (susceptible QTL parent) genome. B) Extra-locus duplications from iF and their mapping locations in the Iinb1 (resistant QTL parent) genome. (PDF) [file pgen.1010570.s004.pdf]

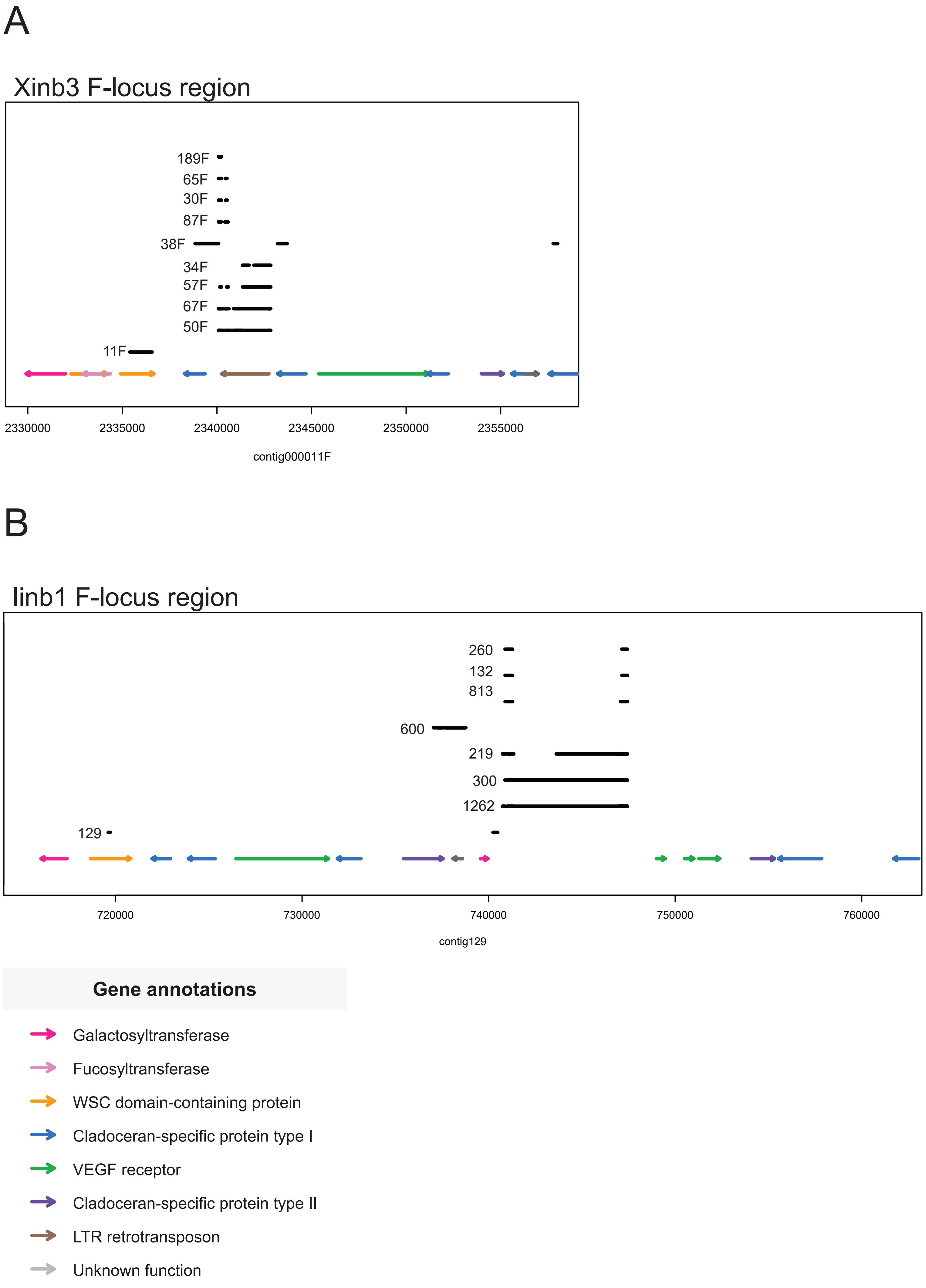
A
Xinb3 F-locus region
189F
65F
30F
87F
38F
34F
57F
67F
50F
11F
2330000
2335000
2340000
2345000
2350000
2355000
contig000011F
B
Iinb1 F-locus region
260
132
813
600
219
300
1262
129
720000
730000
740000
750000
760000
contig129
Gene annotations
Galactosyltransferase
Fucosyltransferase
WSC domain-containing protein
Cladoceran-specific protein type I
VEGF receptor
Cladoceran-specific protein type II
LTR retrotransposon
Unknown function
