# Supplementary material for: QTL study reveals candidate genes underlying host resistance in a Red Queen model system
Source: PLoS Genet. 2023 Feb 2;19(2):e1010570. doi: 10.1371/journal.pgen.1010570 (PMC9894429; doi:10.1371/journal.pgen.1010570)
Supplement: S7 Fig — Boxplots showing gene expression data collected across multiple stressful conditions for a previous study [30], with raw RNA-seq reads from QTL parent clones Xinb3 and Iinb1 mapped to the genome-based transcriptome from clone Iinb1. Gene names are underlined with colors corresponding to functional annotation (see Fig 1). Y-axes from each plot show normalized read counts with a pseudocount of 0.5 added to allow for log-scale plotting. Each plotted point represents a Daphnia magna individual from the respective clone. Box edges indicate first and third quartiles, central line indicates median, and whiskers extend to 1.5 x interquartile range. Asterisks indicate genes that show significant differential expression (non-zero logarithmic fold change in mean expression across all treatments combined) between QTL parent clones Xinb3 (n = 25) and Iinb1(n = 24), with clone Iinb1 as reference after correcting for multiple tests: Benjamini-Hochberg-adjusted p-value < 0.05 (*), < 0.01 (**), < 0.001 (***). Asterisks are colored according to the parent clone which shows higher expression (red = susceptible parent Xinb3; blue = resistant parent Iinb1). Wald test statistics and Benjamini-Hochberg-adjusted p-values are as follows: A) z = -1.44, p = 0.204; B) z = 2.36, p = 0.0312; C) z = -1.11, p = 0.339; D) z = 1.24, p = 0.279; E) z = -4.61, p = 1.19E-05; F) z = 8.51, p = 1.26E-16; G) z = -18.82, p = 2.29e-77; H) z = 4.80, p = 5.01E-06. (PDF) [file pgen.1010570.s008.pdf]

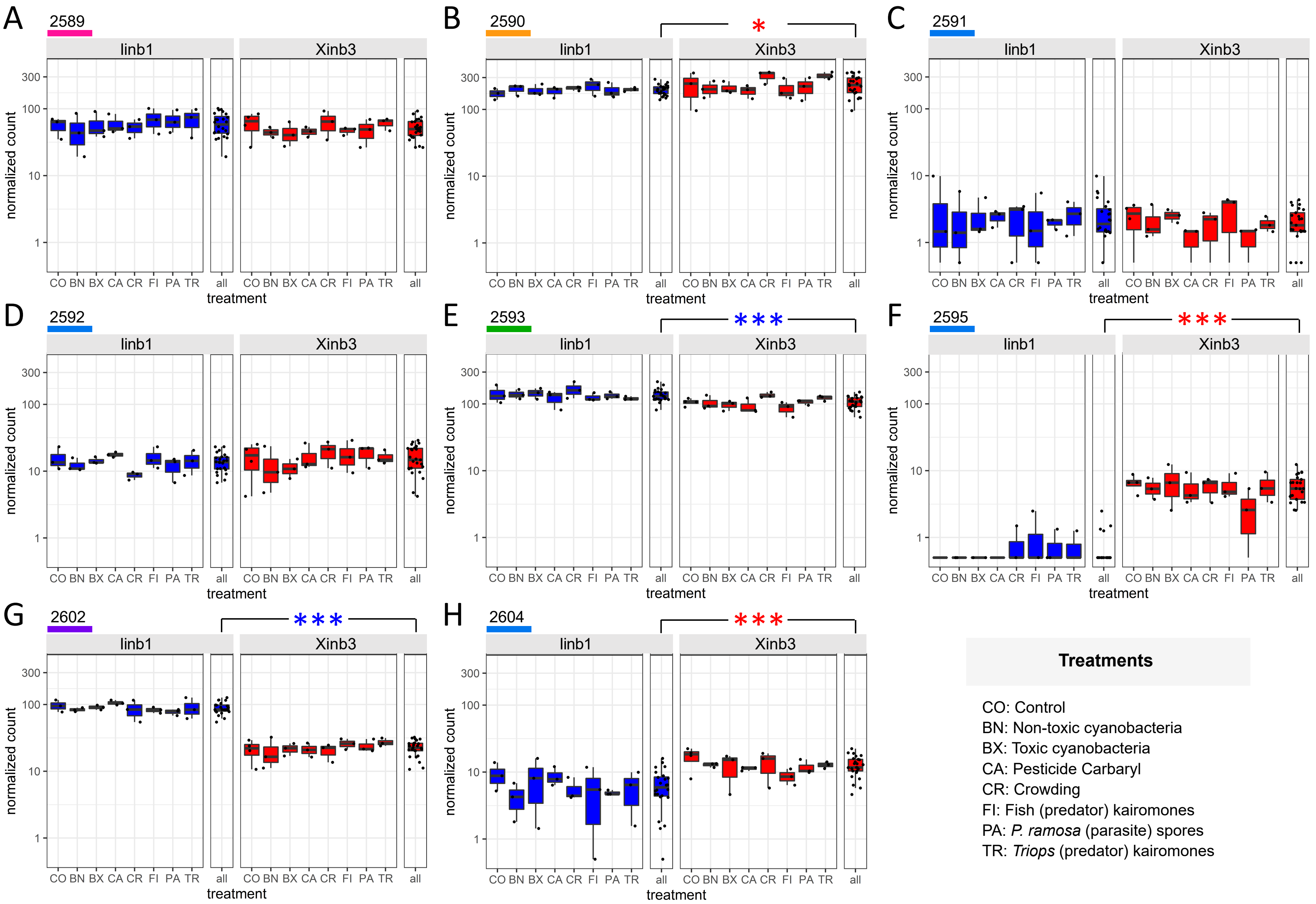
A
2589
B
2590
C
2591
D
2592
E
2593
F
2595
G
2602
H
2604
Iinb1
Xinb3
normalized count
300
100
10
1
CO BN BX CA CR FI PA TR
all
treatment
*
***
Treatments
CO: Control
BN: Non-toxic cyanobacteria
BX: Toxic cyanobacteria
CA: Pesticide Carbaryl
CR: Crowding
FI: Fish (predator) kairomones
PA: P. ramosa (parasite) spores
TR: Triops (predator) kairomones
