# Supplementary material for: QTL study reveals candidate genes underlying host resistance in a Red Queen model system
Source: PLoS Genet. 2023 Feb 2;19(2):e1010570. doi: 10.1371/journal.pgen.1010570 (PMC9894429; doi:10.1371/journal.pgen.1010570)
Supplement: Attachment — Submitted filename: Fredericksen_PlosGen2022_ResponseToReviewers_21112022.docx [file pgen.1010570.s017.docx]

21 November 2022

Dear Editors,

We considered all comments by the reviewers and we believe the study benefited a lot form these inputs. Below is a point-by-point reply to all comments (specific author and editor critiques have been emphasized in bold to enhance readability). We want to thank you and both reviewers for the time invested into reviewing our manuscript. We hope that the revised version of the manuscript finds your agreement.

Sincerely,

Maridel Fredericksen, Ph.D., and co-authors

Summary of reviewer criticisms, provided by the editor:

The primary criticisms are about **putting the current work in proper context in light of your and others' work.** In many respects, much of the present study seems to be framed as the abstract and introduction for a broad review, rather than the focus being on the identification of a very interesting locus, which is the culmination of your efforts. As complimentary as the reviewers are of the work, they are also very critical about the **presentation of the manuscript**, which in current form does not do a good enough job to **highlight the significance of the present study**. Indeed, one of the reviewers felt that, in current form, the study may not rise to the level of significance we desire for PLOS Genetics. I do feel that many of these criticisms can be address by a **serious reworking of the framing of the paper**.

*Thank you for this helpful summary. We have significantly rewritten the abstract, introduction, and discussion sections to reframe the paper. Specifically, we have de-emphasized the broader context of Red Queen coevolution in favor of focusing on the identified resistance locus. We believe this reframing better highlights the contribution of our study in the context of understanding genetic underpinnings of host resistance.*

**Comments to the Authors:**

Reviewer #1: The genetic basis of host-pathogen coevolution is a topic of considerable interest. The system studied here is an important model of coevolution, thanks in a large part to the efforts of this research group. Over recent years they have made progress in understanding this system at a genetic level. The significance of this work is increased by the context of these species being a well-characterised model in the field, and this manuscript adds to this literature. It reports a QTL affecting susceptibility to a bacterial parasite in Daphnia. The QTL contains 13 genes, but the authors are unable to identify which of these confers resistance. The QTL mapping panel, genomes and RNAseq data have been published before, frequently in similar studies using different parasite genotypes. The major new data presented here is therefore the phenotyping.

The main clear result of the paper is that resistance to a new pathogen genotype is controlled by a major-effect Mendelian locus that is different from that controlling resistance to previously studied pathogen genotypes. Furthermore, this locus has numerous rearrangements and gene gains/losses, similar to nearby locus controlling resistance to a different genotype.

The efforts to characterise what the gene might be are weaker, as the genes remain firmly candidates. **A lot of text is devoted to describing these genes and their expression, but this remains circumstantial.** I realise that genetics in this species is challenging, but polymorphisms controlling pathogen resistance have been identified to the gene or nucleotide level in other species. Furthermore, a number of similar QTL studies have been published in this system.

*We have reduced the text describing the gene candidates in the discussion section.*

In this context, it is **unfortunate the authors do not** **acknowledge the literature on genetic polymorphisms affecting host resistance**. For example, the introduction states “the exact identity of the genetic variants responsible for ,resistance/susceptibility polymorphisms segregating in natural populations remains mostly unknown” [and the rest of this paragraph]. The abstract “molecular underpinnings of the genotypic interactions are largely unknown”. **It is poor scholarship to make these statements without citing systems where the statements do not apply, and it detracts from the advances this work might bring to an important model system**. There are many cases where “exact identity of the genetic variant responsible for ,resistance/susceptibility polymorphisms segregating in natural populations” is known. Furthermore, in a number of cases these loci confer resistance only to specific pathogen genotypes. The most prominent cases are perhaps **plant R genes**. In vertebrates, **MHC genes are widely studied**, down to the structural differences underpinning the genotype-specific HIV-MHC epitope binding that affects progression to AIDS. **There are other examples in Drosophila**.

*Thank you for these pointed critiques. We have rewritten the abstract and introduction to reframe the paper in a way that emphasizes discovering genetic polymorphisms affecting host resistance more generally, rather than focusing on naturally coevolving populations. We have cited relevant literature accordingly, including studies on plant R genes, MHC in humans, and Drosophila.*

Minor comments:
Line 97. It would help to be precise about what defines this 50kB region as a supergene? (inversion breakpoints in genome assemblies, LD, lab crosses?)

*Thank you for this suggestion. We have changed the sentence in question so that it specifically references the study that describes the region as a supergene (Bento et al 2017). The sentence now reads, “The F-locus region contains 13 putative genes (positional candidates) and is adjacent to, but distinguished from, a 50-kb Pasteuria-resistance region that was previously described as a supergene (due to apparent lack of recombination), and which contains the A, B, and C loci [16].”*

Line 139. Citation to justify 1.8 LOD drop (I believe a Broman group paper)

*We have removed this passage on the LOD drop (see reply to next comment).*

Line 143. The results and methods need to make clear what exactly was done in the ‘fine mapping’. Is this just scoring more markers? If so, **how is the confidence interval on the QTL location defined**? In the proceeding section a 1.8 LOD drop is used, but there is no mention of the statistics here. I take it this is simply checking for a perfect genotype-phonotype association? It is unclear why this change in statistical approach has occurred – **if this is sufficient here, then why use a LOD drop to analyse a near-identical dataset with fewer markers in the earlier paragraph?** I don’t doubt the results here, but the analysis being conducted and its justification needs clarifying.

*Thank you for pointing out this need for clarification. Indeed, the fine mapping involves adding more markers and checking for a perfect genotype-phenotype association. Therefore, as you point out, a LOD drop is not needed to define the confidence interval of the original QTL. We have removed the passage on the LOD drop and confidence interval, and we have added passages in the results and methods to clarify the fine mapping analysis (lines 128 – 129 and 496 – 498, respectively).*

Figure 2 appears to have been published before by these authors in this journal (slightly different parameters, a new dashed box). Is that correct? Maybe there is a good reason to reproduce the result here with minor changes, but not to acknowledge and cite its earlier incarnation seems surprising. Or maybe I am missing something here?

*Yes, you are correct, and thank you for pointing out this omission. We decided reproduce this figure in order to clearly show the spatial relationship to the newly described locus and compare the structural rearrangements between these two closely located resistance regions. We have revised the figure legend to acknowledge the previously published figure. The last sentence of the figure legend now reads:*

“*This figure has been modified from a previously published figure (Fig 3 in [16]) to specifically compare the location and alignment structure of the ABC supergene and F-locus region.*”

Line 192-249. This could be made considerably more concise – it is hard to see the wood for the tree.

*Indeed, this is a complex section. We removed several sentences that were peripheral to our main message, and we tried to clarify some of the most complex sections. However, we consider this section to be a central part of the study, and the many differences among the two haplotypes do not allow to cut much out, without potentially losing the difference that we are interested in.*

Figure 4. Why not just report the ‘all’ results in the main text? There are lots of apparently irrelevant treatments, making this plot rather complex and hard to read. The sudden appearance of a plot about fish predation is rather befuddling!

*As requested, we have modified figure 4 to only include the “all” results. We have kept the previous figure in the supplement (S6_Figure) so that readers can evaluate the consistency in expression across treatments.*

Line 313-325. This belongs in the Discussion.

*As requested, we have moved this paragraph to the Discussion.*

The Discussion **spends much time repeating results in this and other papers**. It could be made **more concise, focussing on broader context to increase impact**. The manuscript also begins by stating the importance of this work to our understanding of the process of coevolution. **Is there anything to say on this?** I understand this to be one of the few species where the matching alleles model applies – **might these results suggest this results from tightly linked genes following the gene-for-gene system model?**

*At this point, we cannot make conclusions regarding the mode of coevolution operating in this system as a whole. The matching-allele model was shown to apply in relation to two* P. ramosa *genotypes so far. However, in order to understand whether the matching-allele model or gene-for-gene model applies at other loci and in relation to overall resistance to* P. ramosa, *we need to identify the genes in question. This study is a step in this direction. We have significantly rewritten and reduced the Discussion section to more clearly emphasize these points regarding coevolution and to focus on the broader context of the work.*

Three levels of subheadings does not add clarity.

*Thank you for this feedback. We have modified the text to include only the main section headings (e.g. Results, Discussion) and one level of subheadings, and we feel this restructuring improves readability.*

Embedding legends and figures in the text (as opposed to 3 locations) at initial submission is helpful to a reviewer with a small laptop screen…

*We empathize with the reviewer’s sentiments but followed the submission requirements of PLOS Genetics.*

Reviewer #2: In this manuscript the authors use the Daphnia-Pasteuria system to identify and map an additional genomic region affecting bacterial infectivity in the system. The authors identify eight candidate genes within the region with the potential to explain the differences in bacterial spore attachment between the parents used for QTL mapping. Overall, this work is incredibly thorough and adds an important piece to characterizing the basis for host resistance to Pasteuria infection. Further, the natural context and ecological dynamics within the system make this a very promising system to phenotypically and genotypically characterize coevolutionary dynamics in the wild.

*Thank you very much for these encouraging words.*

Overall, I think this is an important study within the field and for the system. However, I **believe this manuscript struggles somewhat to stand on its own**. I fully agree that this system has great potential to characterize the Red Queen in action. But, **this study does not directly investigate coevolution.** Rather, the focus of this work is mapping the F locus and identifying candidate genes. While I generally agree with everything the authors state about the system relative to the Red Queen, **I think the paper could be re-framed somewhat to highlight the work that is presented rather than the potential of the system as a whole**. It is currently unclear to me how the work presented here connects to the Red Queen, other than adding an additional locus to track. Further, **it is not clear what adding an additional locus might mean for testing the Red Queen.**

*Thank you for these insightful critiques and suggestions. We have revised the abstract, introduction, and discussion sections* *to reframe the paper in a way that de-emphasizes the Red Queen and focuses on the findings from this study and their contribution to the literature on the genetic underpinnings of host resistance.*

It seems to me that identification of the F locus is the most significant finding within this manuscript. However, the **F locus is not specifically mentioned in the abstract (or the title).**

*Thank you for this suggestion. We now specifically mention the F locus in the abstract.*

I find it quite interesting that **what seemed a relatively straightforward host-pathogen interaction has seemingly become more and more complex with further investigation. This is noteworthy and would seem worth highlighting**.

*This is a good point. We have added a discussion of the implications of adding the F locus and the increasing complexity of this host – parasite interaction (lines 326 – 336).*

This is merely a suggestion, but an **introduction more focused on the evolution and genetic architecture of host resistance and a discussion more focused on the implications of finding this additional locus may allow the manuscript to stand on its own more effectively.**

*Thank you for these suggestions. We have revised the introduction and discussion sections to emphasize these points.*
